# The ParlLawSpeech Parliamentary Text Data Collection > 3 Million Parliamentary Bills, Laws, and Speeches from 8 Legislatures

**DOI:** 10.1038/s41597-026-08335-4

**Published:** 2026-09-19

**Authors:** Jan Schwalbach, Lukas Hetzer, Sven-Oliver Proksch, Christian Rauh, Miklós Sebők

**Affiliations:** 1 GESIS - Leibniz Institute for the Social Sciences, Cologne, Germany; 2 University of Cologne, Cologne, Germany; 3 WZB Berlin Social Science Center, Berlin, Germany; 4 University of Potsdam, Potsdam, Germany; 5 Democracy Institute, Central European University, Budapest, Hungary; 6 Syracuse University, Syracuse, NY USA

## Abstract

Parliamentary texts are central sources for studying democratic representation, policy-making, and institutional decision-making, and they also constitute a highly valuable domain for natural language processing research. Despite their importance, access to comprehensive, clean, and machine-readable parliamentary corpora remains limited. This paper introduces the ParlLawSpeech (PLS) collection, a large-scale, curated corpus comprising more than three million documents from eight European parliaments, each covering over two decades. PLS extends existing parliamentary text resources in two key ways. First, it integrates full texts and metadata not only for parliamentary speeches but also for legislative bills and finally adopted laws. Second, it provides systematic data linkage across these document types, enabling researchers to trace the full parliamentary decision-making process from draft legislation through debate to adoption. Covering seven national parliaments and the European Parliament, PLS offers a uniquely rich resource for political science and advanced NLP research.

## Background & Summary

Parliaments are key institutions for democratic societies. Documents emerging from parliamentary processes encode dense and detailed information on societal challenges, political agendas, representation, policy-making, and institutional decision-making. Thus, political and social scientists increasingly rely on computational text analysis for extracting and analysing such information from parliamentary records at scale. For example, political scientists have used parliamentary corpora to study patterns of debate participation, party discipline, and representation^1^, to track polarization over time^2^, or to measure emotion and rhetoric in legislative debate^3,4^, while sociologists have drawn on them to trace long-run semantic shifts in expressed solidarity toward women and migrants, for instance^5^.

In addition to their substantive importance, parliamentary texts also represent a highly valuable domain for research in natural language processing (NLP) and computational linguistics. Parliamentary debates constitute a highly structured form of institutional discourse, produced under formal procedures by identifiable speakers and authors with stable roles and affiliations. These characteristics make parliamentary speech corpora particularly suitable for developing and evaluating NLP methods that require discourse structure and rich metadata, including cross-genre topic modelling^6^, argument mining^7^ and stance detection^8^, as well as contextual document classification^9^ and advanced summarization tasks^10,11^. Moreover, the longitudinal nature of parliamentary records and their availability across multiple languages provide opportunities for research on semantic change and diachronic representation learning^12^, as well as cross-lingual modelling^13^.

Parliamentary corpora, therefore, constitute an important source for substantive analysis of societal and political challenges and state-of-the-art NLP research. Despite their significance, parliamentary text collections are oftentimes difficult to obtain in comprehensive, clean, consistent, and machine-readable formats, posing a significant challenge for applied research^14,15^. High-quality single-country collections of parliamentary speeches have recently been published in *Scientific Data*^16–18^. Several large projects also offer multi-country collections of such speeches, most notably *ParlaMint*^19^ in the linguistics domain and *ParlSpeech*^20^ or *Parl-EE*^21^ in the domain of political science, while projects like *IOParlSpeech*^22^ or *ParlText*^23^ build on and add to existing corpora.

This data descriptor extends these efforts and introduces the *ParlLawSpeech* (PLS) collection, developed as part of Work Package 5 of the OPTED project (Observatory of Political Texts in European Democracies; https://opted.eu), which provides researchers with direct access to a complete and well-curated set of more than three million documents from eight European parliaments covering time spans of more than two decades each. Besides extending the coverage of readily usable parliamentary text corpora, PLS introduces innovations in two other key areas. First, PLS not only contains parliamentary speeches but also full texts and metadata for parliamentary bills and adopted laws. Second, PLS offers data linkage across different document types in a way that is typically not even offered by the institutional repositories themselves^24^. These two innovations allow researchers to systematically map and combine different parliamentary outputs, thereby enabling them to address research questions spanning the full process of parliamentary decision-making, from draft legislation and plenary debates to the contents of the finally adopted laws. In sum, PLS offers machine-readable and linkable full-text vectors of parliamentary speeches, bills, and laws, as well as relevant metadata for seven European countries (Austria, Czechia, Croatia, Denmark, Germany, Hungary, and Spain) and the supranational European Parliament (EP).

## Methods

The datasets available in PLS build on official repositories and archives and were enhanced by customized data collection approaches which we each individually validated in order to provide a ready-for-research format. In general, five generic steps were applied for each dataset construction that we describe in detail below. Before these steps were applied, we had to define what counts as a speech, bill, and law for each of our cases. We decided to focus on the politically more relevant lower houses in countries with a bicameral system. Tables 1–3 list the exact inclusion criteria for all parliaments and every data type.

**Table 1:** Inclusion criteria for speech documents.

Country	Speech selection criterion	Document types
Austria	Documents classified as “NRSITZ” (Nationalrat – Plenarsitzung) on the webpage of the Austrian parliament	3 document types: Sitzung, Sondersitzung, Zuweisungssitzung
Croatia	Documents classified as “Rasprave“ on the webpage of the Croatian parliament	No further subdivision into document types
Czech Rep.	Documents classified as “stenoprotokoly” (stenographic records) in the digital library of the Czech parliament for the respective parliamentary cycle	No further subdivision into document types
Denmark	Documents classified as “Referater” on the webpage of the Danish parliament	No further subdivision into document types
EP	Documents classified as “verbatim reports” on the webpage of the European parliament	No further subdivision into document types
Germany	Documents classified as “Plenarprotokolle” on the webpage of the German parliament	No further subdivision into document types
Hungary	Documents classified as “felszólalás” (a speech) in the “országgyűlési napló” (verbatim diary) of the Hungarian parliament	No further subdivision into document types
Spain	Documents classified as “Diario de Sesiones” on the webpage of the Spanish parliament	2 document types: Pleno, Diputación Permanente

**Table 2:** Inclusion criteria for bill documents.

Country	Bill selection criterion	Document types
Austria	Documents classified as “Gesetzesinitiativen” (legislative initiative) on the webpage of the Austrian parliament	9 document types. Most frequent: Selbstständiger Antrag, Regierungsvorlage, Regierungsvorlage Staatsvertrag (together >94%)
Croatia	Documents classified as “PZ” (proposal of an act) or “PZE” (proposal of an act with EU compliance) on the webpage of the Croatian parliament	2 document types: hitni, redovni
Czech Rep.	Documents classified as “návrh zákona” (a draft law) on the webpage of the Czech parliament	No further subdivision into document types
Denmark	Documents classified as “Lovforslag” (legislative proposal) on the webpage of the Danish parliament	No further subdivision into document types
EP	Documents classified as “Lawmaking procedure” on the EUR-Lex database	29 document types. Most frequent: Proposal for a regulation, proposal for a decision without addressee, proposal for a decision, proposal for a directive (together >82%)
Germany	Documents classified as “Bundestag-Drucksache” (printed document) AND “Gesetze, Rechtsnormen” (laws, legal norms) AND “Gesetzgebung” (legislation) on the webpage of the German parliament	No further subdivision into document types
Hungary	Documents classified as “törvényjavaslat” (bill) in the parliamentary documents register of the Hungarian parliament	No further subdivision into document types
Spain	Documents classified as “Función legislativa” (legislative function) on the webpage of the Spanish parliament	9 document types. Most frequent: “122” (Proposición de Ley), “121” (Proyecto de Ley), “130” (Real Decreto-ley) (together: >86%)

**Table 3:** Inclusion criteria for law documents.

Country	Law selection criterion	Document types
Austria	Documents classified as “Bundesgesetze” (federal laws) or “Bundesverfassungsgesetze” (federal constitutional laws) on the webpage “Rechtsinformationssystem des Bundes” (Federal Legal Information System)	2 document types: Bundesgesetze, Bundesverfassungsgesetze
Croatia	Documents classified as “Objava” (publication) linked on the webpage of the Croatian parliament to the “Narodne Novine” (Official Gazette) webpage	No further subdivision into document types
Czech Rep.	Documents classified as “zákon” (act) in the “Sbírka zákonů” (Collection of Laws) webpage	No further subdivision into document types
Denmark	Documents classified as “Lov” (law) or “Ændringslov“ (amendment act) on the “Retsinformation” (Legal Information) webpage	2 document types: Lov, Ændringslov
EP	Documents classified as “adopted act” linked on the EUR-Lex webpage to the respective lawmaking procedures	19 document types. Most frequent: Regulation, decision, directive ( <91%)
Germany	Documents classified as “Bundesgesetze” (federal laws) on the webpage of the Federal Gazette (Bundesgesetzblatt)	No further subdivision into document types
Hungary	Documents classified as “törvény” (act) in the “Nemzeti Jogszabálytár” (National Legislation Database)	No further subdivision into document types
Spain	Documents classified as “leyes aprobadas” (laws passed) on the webpage of the Senado de España	4 document types: Ley, Ley Orgánica, Real Decreto-Ley, Real Decreto Legislativo

We included all stenographic records from full plenary sessions (both normal and emergency sessions if applicable) as the basis for our speech data. A single speech is then defined as all words a listed speaker (including non-MP speaker) speaks until the next speech starts. This next speech may be an interruption (e.g., by the chair) leading to a continuation of the original speech in the speech after the interruption. Whether and if so, how interruptions are recorded in the stenographic records depends on the parliament. For the bill datasets, we included all documents that were listed as legislative proposals on the respective parliamentary website. We used this inclusive approach to allow for as many research questions as possible.

Finally, the law documents in most cases are a subset of the bill proposals if they are passed in their final version. However, there are two exceptions: 1) In rare cases, bill proposals are split in the legislative process resulting in more than one law document being linked to a bill document. 2) In the case of Spain, Real Decretos are included that have the force of a law that the government may issue in cases of exceptional and urgent necessity without prior approval from parliament.

The first task to create the datasets involved identifying the most suitable data source for the three different data types. In most cases, only one source existed, usually the official webpage of the respective parliament for debate protocols and sometimes for bills, as well as a dedicated archive of laws and, in some cases, bills. When multiple sources existed, we always opted for the one offering the longest temporal span of consistent and accessible document types^14^. Table 4 provides an overview of data sources and the respective source file formats.

**Table 4:** Data Sources and Formats.

Parliament	Data Source	Data Format
Austria - Nationalrat	https://www.parlament.gv.at/gegenstand/XXVII/NRSITZ/ (speeches) https://www.parlament.gv.at/recherchieren/gegenstaende/gesetzesinitiativen (bills) https://www.ris.bka.gv.at/Bgbl-Auth/ (for laws 2004-) https://www.ris.bka.gv.at/Bgbl-Pdf/ (for laws −2004)	html (bills) & pdf (speeches, laws)
Croatia - Sabor	https://edoc.sabor.hr/Fonogrami.aspx (speeches) https://edoc.sabor.hr/Akti.aspx (bills) https://narodne-novine.nn.hr/search.aspx (laws)	html (speeches) & pdf (bills, laws)
Czech Republic - Poslanecká sněmovna	https://www.psp.cz/eknih/2013ps/stenprot/index.htm (speeches, e.g. for 2013) https://www.psp.cz/sqw/sntisk.sqw (bills) https://aplikace.mvcr.cz/sbirka-zakonu/ (laws, currently working version: https://e-sbirka.gov.cz/)	html (speeches, laws) & pdf (bills)
Denmark - Folketing	https://www.ft.dk/da/dokumenter/dokumentlister/referater (speeches) https://www.ft.dk/da/dokumenter/dokumentlister/lovforslag (bills) https://www.retsinformation.dk/documents?dt=10&dt=20&o=40&ps=100&t=LOV%20nr (laws)	html (speeches, laws) & pdf (bills)
EU – European Parliament	https://www.europarl.europa.eu/plenary/en/debates-video.html (speeches) https://eur-lex.europa.eu/advanced-search-form.html (bills & laws)	html (all data)
Germany - Bundestag	https://www.bundestag.de/dokumente/protokolle (speeches) https://dip.bundestag.de/erweiterte-suche (bills) https://www.bgbl.de/xaver/bgbl/start.xav#/text/I_2022_57_inhaltsverz?_ts=1780997724998 (laws)	pdf (all data)
Hungary - Országgyűlés	https://www.parlament.hu/web/guest/orszaggyulesi-naplo (speeches) https://www.parlament.hu/iromanyok-lekerdezese (bills, currently working link: https://www.parlament.hu/web/guest/iromanyok-m) https://njt.hu/ (laws)	html (speeches) & pdf (bills, laws)
Spain – Congreso de los Diputados	https://www.congreso.es/es/busqueda-de-publicaciones (speeches) https://www.congreso.es/es/busqueda-de-iniciativas (bills) https://www.senado.es/web/actividadparlamentaria/actualidad/leyes/aprobadas/index.html (laws)	html (all data)

In the next step, we set up the collection of the original source documents as an extensive web-scraping exercise along with scripts in the R language^25^, customised specifically to each individual institutional archive for each text type in each country. The complexity of these web scrapers varies immensely and depends on the type of data and the organization of the respective website. This ranges from simple bulk downloads of documents in rare cases to the complexity of individual subpages with individual speeches from dynamic websites, which require automated browsing to save the respective speeches and metadata. In these efforts, we most notably relied on the R packages *RSelenium*^26^, *XML*^27^, and *rvest*^28^.

Once the complete set of documents for the longest available period for each of the three document types and parliaments was downloaded and locally stored, we then split the documents and extracted the document texts. Scripts extracted the speech, bill, and law text for each document, a task that was again highly customized to the specific parliamentary records as it requires careful conceptual unitizing as well as acquaintance with the respective parliamentary and language context^14,29^. In general, we minimized the amount of pre-processing of the document texts themselves and provide clean strings so as to give researchers the greatest possible flexibility in preparing and processing the text for their specific research demands. In the case of bill texts, this also means, for example, keeping text sections that are not part of the core paragraphs of the proposal in the text variable (e.g., explanation of the motivation). In the case of speeches, interruptions (if recorded in the protocols) were also retained, and, where possible, a second variable was created with the text data without interruptions.

As in the previous steps, the level of complexity and the risk of errors vary, primarily depending on the document type and any changes to the document structure over time. In the easiest cases, the speech, bill, or law text in a html document is tagged as such (e.g., speech documents from the Croatian parliament). In more challenging cases, this requires the definition of various regular expression patterns to address text boxes in pdf documents that sometimes even span over several columns (e.g., speech documents from the German parliament). Useful R packages that we used for these kinds of tasks include *stringr*^30^, *stringi*^31^, *glue*^32^ as well as *pdftools*^33^.

Extracting the relevant metadata as the next step focused on information details provided along the raw document text (e.g., speaker names at the beginning of a speech or title of a bill at the beginning of a bill text). The section *Data Record* below lists all derived metadata. In some cases, metadata was added from sources other than the original documents alone. For example, in the case of the Spanish speech documents, the name of the speaker’s party is not included (only the name of the parliamentary fraction). This information was obtained additionally from a different subpage of the parliamentary website. Checking back with country experts and defining what fits the categories was important to prevent misclassification.

Linking document types and creating a common identifier constituted the final step. We first inspected the bill archive of each parliament to identify the locally unique way of referring to a bill – typically a local numbering system encoding legislative periods, dates, and running numbers of bills therein. In some countries, these are unique, e.g., in Germany: two digits indicating the legislative period followed by “/“ followed by a 6-digit number. In cases where this pattern is repeated annually (e.g., Spain: three digits indicating the type of bill proposal followed by “/“, followed by 6 digits) or per legislative term, the identifier had to be extended with a unique temporal tag. In all stenographic protocols from the parliaments that we included, the reference numbers of bills are used in the agenda titles if a certain bill is discussed. This allowed us to match the identifiers in the respective agenda documents for plenary debates and add the bill identifier to all speeches given under the matching agenda item. In some cases, the respective websites that included the bill documents also contained metadata about the days when the bills were discussed in parliament. This information served as additional validation material if available.

For most parliament, the linkage of the bills to the respective laws was easier as in many cases the respective law (if passed) was already linked to a bill proposal on the respective website. In the remaining cases, we inspected the respective archive of laws to learn how bills are referred to in either the laws’ texts or, preferably, the metadata provided by the archive. Once the pattern was established, we matched laws and bills with a respective string-matching procedure.

As an additional service to the research community, we also auto-translated the speech corpora of the European and the Hungarian parliaments and provide English-language versions of these speeches as part of the data release (see *Data Record* below). Within our budgetary constraints, these two parliaments were prioritized because the European Parliament is the only genuinely supranational, multilingual legislature in Europe (and stopped providing own official English translations in 2012), while the Hungarian Országgyűlés combines substantive relevance for research on democratic backsliding with a language rarely read by the international scholarly community. The translations were implemented using the Google Cloud Translation API (Neural Machine Translation model, “nmt”), which was selected because of its large language-pair availability needed for the multilingual EP corpus, its API-based scalability, and - importantly - prior validation of this translation model for standard political science applications of text analyses^34,35^.

For the European Parliament, where speakers frequently switch languages mid-speech, texts were first tokenized to the sentence level; local language detection (fastText, supported by CLD2/CLD3 cross-checks) identified non-English sentences, which were submitted individually to the API (automatic source-language detection) and subsequently re-aggregated to the speech level, yielding 240,932 (partially) translated speeches out of 509,649. For the Hungarian parliament, where multilingualism was negligible, translation was performed at the full-speech level (487,877 speeches); four speeches exceeding the API’s per-request character quota were excluded from translation and retained in the original language. These translations - costing around 25k € - in total are provided as an additional service to enhance data access for scholars, but we advise task-specific validation for advanced research questions and retain the original language records in the data throughout^36,37^ (see also *Techncial Validation* and *Code Availability* below).

## Data Record

All datasets (DOI: https://doi.org/10.7802/2824) are publicly and permanently available from the GESIS archive at https://search.gesis.org/research_data/SDN-10.7802-2824^38^. All datasets can be downloaded freely under the Open Access category from GESIS. The speeches, bills, and laws datasets are provided in.RDS formats for compactness and easier use in the open-source R environment (Corpus_[speeches/bills/laws]_[country].RDS), from where they can be easily transferred to any desired format. All variables, if not indicated otherwise, are UTF-8 encoded. In the following, we provide short descriptions of the variables in the three corpora. This consists of a “core” set of variables available for each document type across countries, as well as “additional” metadata that were only available for some sources but should not be dropped, as they are potentially useful for specific research projects in the future. The most important novelty of the PLS datasets is the unique identifier, which enables linking documents across the three corpora. This procedure_ID, found in the speeches, bills, and laws datasets alike, is based on the conventions of the respective countries’ parliamentary websites or other legislative databases. With the help of this ID, researchers can track which bills became which laws and if there were any speeches given regarding them during the legislative process.

### Speech corpora

The first set of corpora is composed of the parliamentary speeches in clean text formats (text) alongside their respective metadata (see Table 5 for an overview of all included core variables). The date on which the speech was given (in YYYY-MM-DD format) is included in the dataset, ensuring accurate filtration of relevant texts for researchers. The speaker variable indicates the name of the person who gave the respective plenary speech, while the party column holds the speaker’s party affiliation (in the case of the European Parliament, the variable refers to the party group and not to the national party affiliation). These can be useful for identifying trends in parliamentary parties’ activity, as well as finding topics or themes specific to them. In the case of the Czech corpus the party affiliation as well as the party support of non-MP speakers is included in this variable.

**Table 5:** Core variables of the speech corpora.

Speeches corpora – core variables *Data on plenary speeches in the respective parliament (in chronological order across and within sessions)*		
Variable	Description	Variable Format
speaker	Name of the person who gave the plenary speech	chr
text	Full text of the respective speech	chr
party	Party or partisan factor of the speaker	chr
date	The date on which the speech was held	date
agenda	The agenda item under which the speech was given	chr
speechnumber	Unique ID per session reflecting the chronological order	num
procedure_ID	The unique PLS identifier linking speeches, bills, and laws	chr
partyfacts_ID	ID that facilitates linkage to core parties in the PartyFacts dataset	chr
period	Legislative periods included in the corpus	chr

The agenda items under which the speeches were given are available for all legislatures, holding a string with the title of the respective agenda points. This variable provides context for the speeches, helping identify themes or facilitate comparison between countries. Speechnumbers are IDs reflecting the chronological order of the speeches within the given session, providing a way to sort and reference speeches conveniently. This variable is numerical, except for the Hungarian dataset: Due to the specific data structure of the Hungarian parliamentary website, in some cases, several speeches are assigned to one speaker in a single row. In this case, the respective variable indicates the number of speeches (e.g. “4–6”). Finally, the variable partyfacts_ID makes it possible to link a speaker’s party affiliation to parties in the PartyFacts dataset^39^. The legislative period derived from the dates is included in most cases, either holding the electoral cycle (as in the case of the Hungarian data) or a unique ID of the legislative period (as seen in the Austrian corpus).

Beyond this core set of variables, we include several additional variables that might be useful for specific applications if the respective legislature’s official sources provided them (see Table 6). In some cases, the session variable specifies the session number within the legislative period as counted by the legislature (this is especially relevant when more than one session was held per day). The chair variable (logical TRUE/FALSE values) indicates whether the speaker was the formal chair of the given session. It is useful for filtering relevant speeches, as the ones given by the chairs usually only hold the agenda points and the names of the speakers. Links to the original speeches are provided in many datasets, as they are essential for traceability and the overall reliability of the corpora.

**Table 6:** Additional variables of the speech corpora.

Speeches corpora – additional variables *Data on plenary speeches in the respective parliament (in chronological order across and within sessions)*		
Variable	Description (parliaments included)	Variable Format
session	Session number within the legislative period as counted by the legislature (AU, HR, HU, ES)	chr
chair	Indicates whether the speaker was the chair of the given session (AU, CZ, DK, EP, D, HU, ES)	logical
link	Link to the original speeches on the parliamentary website (AU, HR, CZ, DK, EP, HU)	chr
text_raw	Uncleaned version of the speech text including e.g. records of interjections or applause (AU, D, ES)	chr
chancellor; minister	Indicator whether the speaker is chancellor or minister in the German data (D)	logical
schriftführer	Indicator whether the speaker holds the position “Schriftführer” in the Austrian data (AU)	logical
position	Indicator for important positions (e.g., minister) of speakers in the Czech data (CZ)	chr
MEP; commision	Indicator whether the speaker is a MEP or commissioner in the EP data (EP)	logical
grupo	Specifying the parliamentary fraction of a MP in the Spanish parliament (ES)	chr
written	a logical variable holding whether the speech was submitted in written form (EP)	logical
multispeaker	If a written speech was submitted by more than one person, this is indicated with multispeaker (EP)	logical
translatedText	English translations of the speech column (EP, HU)	chr
translationInSpeech	Indicates cases with translation (EP, HU)	logical

Raw texts (text_raw) were retained in the corpora in certain cases to make the few text cleaning and preprocessing steps reproducible and traceable, allowing, among other things, the identification of potential errors in our light-weight procedures. Furthermore, these raw texts and speaker names can contain additional information, such as spontaneous interventions during a speech or additional speaker information, such as titles. There is also supplemental information on the speakers in some datasets, such as the chancellor, minister, or schriftführer variables, each indicating different positions in the parliaments. In the case of the Czech data, there is a general position variable that indicates all labelled positions and a variable that indicates whether a speaker is MP.

The Spanish grupo column specifies if the speaker is part of a certain parliamentary fraction. The EP dataset has numerous unique columns: MEP and commission indicate if the speaker is a member of the European Parliament or the European Commission, respectively. Written is a logical variable holding whether the speech was submitted in written form. These written contributions to a debate in the European parliament are not delivered as a spoken speech during a parliamentary session but instead submitted to the debate in written form to be added to the protocol. In these cases of written speeches, it is possible that the text was submitted by more than one person, which is indicated with the variable multispeaker.

The Hungarian and the EP dataset contain a variable translatedText with English translations of the speech column. As not all speeches in the EP dataset were translated due to the document availability during the translation process, the variable translationInSpeech indicates cases with translation.

### Bill corpora

The second set of corpora holds the cleaned full-text vectors of bills (bill_text), as well as their titles (title_bill) and relevant metadata (see Table 7 for an overview of all included core variables). The legislative body’s final decision regarding the bill is stored in the column status, making the comparison of, for example, rejected and passed bills possible. Finally, similarly to the speeches’ dataset, the initiation_date (in YYYY-MM-DD format) pinpoints the introduction date of each bill.

**Table 7:** Core variables of the bills corpora.

Bills corpora – core variables *Data on legislative bills tabled in the respective parliament*		
Variable	Description	Variable Format
title_bill	The title of the bill	chr
bill_text	Full text of the bill	chr
status	The final decision of the legislative body regarding the bill	chr
procedure_ID	The unique PLS identifier linking speeches, bills, and laws	chr
initiation_date	The day on which the bill was introduced	date

There are also several potentially useful additional variables in the bills corpora across the legislatures (see Table 8). The bill_ID is an identification number of the bill, following the conventions of the respective parliaments and enabling researchers to match bills with external data sources beyond the PLS corpora. The period column clarifies the legislative period within which the bills were introduced. Tracking the legislative activity of different members of the parliament (and indirectly of parties, for instance), the initiator of the given bills is also included. Bill types are specified in the bill_type column, categorising them according to the respective parliaments’ systems (such as by the initiators in the Spanish case). In the case of Croatia, bill proposals stemming from EU law are indicated with the variable bill_EU.

**Table 8:** Additional variables of the bills corpora.

Bills corpora — additional variables Data on legislative bills tabled in the respective parliament		
Variable	Description (parliaments included)	Variable Format
bill_ID	Identification number of the bill, following the conventions of the respective parliaments (AU, HR, DK, D, ES)	chr
period	Legislative period within which the bills was introduced (AU, HR, CZ, DK, D, ES)	chr
initiator	Initiator (e.g., ministry or party) of a bill (AU, HR, DK, EP, D, HU, ES)	chr
bill_type	Bill type according to the respective parliaments’ systems (AU, HR, EP, ES)	chr
bill_EU	Indicates bill proposals stemming from EU law (HR)	logical
voting_result	Indicates how many MPs voted for and against a bill (HR)	chr
amendments	Number of amendments proposed in the legislative process (HR)	chr
committee	Indicates the committees for consideration a bill was referred to (AU, HR, DK, D, ES)	chr
bill_link	Link to the original bill on the parliamentary website (AU, HR, CZ, DK, D, EP, HU)	chr
CELEX; procedure_number; proposal_number	Identifier assigned to each respective document on the European parliament website (EP)	chr

Information regarding the legislative process (such as debates and decisions on the bills introduced) is also included in certain cases. Voting_result & amendments provide additional angles for researchers, possibly aiding in the recreation of comparing the unfolding of the legislative processes. The former variable indicates how many MPs voted for and against a bill, and the latter indicates the number of amendments proposed in the legislative process. The committee variable indicates the committees a bill was referred to for consideration, offering insight into the black box of governance. The bill_link for the bill texts is also included in some cases. The unique characteristics of EP bills also appear in the columns: CELEX, procedure_number, and proposal_number hold an identifier assigned to each respective document (instead of the variable bill_ID). Procedure_type specifies the type of legislative procedure under which the bill falls.

### Law corpora

The third set of corpora, comprising full-text vectors of laws (law_text) and their titles (title_law), is completed with metadata and presented in Table 9. The variables in these datasets resemble those of the speeches and bills corpora: besides the procedure_ID, adoption_date provides a picture of the legislative timeline. It is noteworthy that the length and content of bill and law titles may vary both across and within legislatures due to differences in legislative practices or drafting conventions.

**Table 9:** Core variables in the laws corpora.

Laws corpora – core variables *Data on laws finally adopted by the respective parliament*		
Variable	Description (parliaments included)	Variable Format
title_law	The title of the law	chr
law_text	The full text of the law	chr
adoption_date	The day on which the law was published	date
procedure_ID	The unique PLS identifier linking speeches, bills, and laws	chr

The unique and stable columns across these law corpora are also enriched with several useful additions, such as the legislative period, available for researchers concentrating on wider horizons (see Table 10). Law_IDs cater for easier identification when matching with external data, while the law_links are provided for traceability. The EP’s law_type variable is analogous to the bill_type variable of the bill dataset.

**Table 10:** Additional variables in the laws corpora.

Laws corpora – additional variables *Data on laws finally adopted by the respective parliament*		
Variable	Description	Variable Format
period	Legislative period corresponding to the law (AU, CZ, DK, HU)	chr
law_ID	Identification number of the law, following the conventions of the respective parliaments (AU, CZ, DK, D, HU, ES)	chr
law_link	Link to the original law on the parliamentary website (AU, HR, CZ, DK, D, EP, ES)	chr
law_type	Law type according to the respective parliaments’ systems (EP)	chr

## Data Overview

Table 11 summarises the descriptive statistics of the three corpora available for each of the eight parliaments. PLS encompasses at least ten years of data for all document types and parliaments, but the time periods covered vary across legislatures due to availability in the underlying institutional archives of each parliament and the intention to include only completed legislative periods, with the year column denoting either calendar years or the respective government cycles. Thus, minor differences in the time frames can be attributed to the fact that the classifications of speeches, bills, and laws in legislative periods do not entirely overlap.

**Table 11:** Descriptive statistics of the three corpora per legislature.

Country	Data	Count	Variables	Time frame
**Austria**	*Laws*	3030	7	1996–2020
	*Bills*	5926	11	1996–2020
	*Speeches*	204881	14	1996–2019
**Czech Republic**	*Laws*	844	7	2013–2023
	*Bills*	2127	6	2013–2023
	*Speeches*	192979	13	2013–2023
**Croatia**	*Laws*	2972	5	2003–2020
	*Bills*	3676	14	2003–2020
	*Speeches*	405260	11	2003–2020
**Denmark**	*Laws*	3220	7	2007–2022
	*Bills*	3615	10	2007–2022
	*Speeches*	716807	9	2007–2022
**EP**	*Laws*	10554	6	1994–2024
	*Bills*	14105	11	1994–2024
	*Speeches*	574119	17	1999–2024
**Germany**	*Laws*	1638	6	2009–2021
	*Bills*	2445	10	2009–2021
	*Speeches*	191932	13	2009–2021
**Hungary**	*Laws*	4303	6	1994–2022
	*Bills*	7500	7	1994–2022
	*Speeches*	452645	13	1994–2022
**Spain**	*Laws*	1563	6	1996–2023
	*Bills*	4188	10	1996–2023
	*Speeches*	318576	13	1996–2023

The country selection of PLS originally follows two main rationales derived from a political science background: First, we aimed to maximise comparative value by providing data on legislatures across different political systems, ages, and regions of the European Union (including the European Parliament). Second, we built on already existing precursors (especially *ParlSpeech*) to minimise adaptation costs while matching the necessary language knowledge available in our team^14^.

The datasets provided in PLS are as comprehensive as the underlying institutional archives within the period covered. That is, we collected all full texts and metadata for speeches, bills, and laws that each institutional online archive contains (leading to missing values in cases where data is missing or incorrect on the respective website). To address the lack of APIs and common identifiers across individual national archives, particularly for linking speeches, bills, and laws, we conducted a detailed analysis before developing our custom automation tools.

**Fig. 1:**
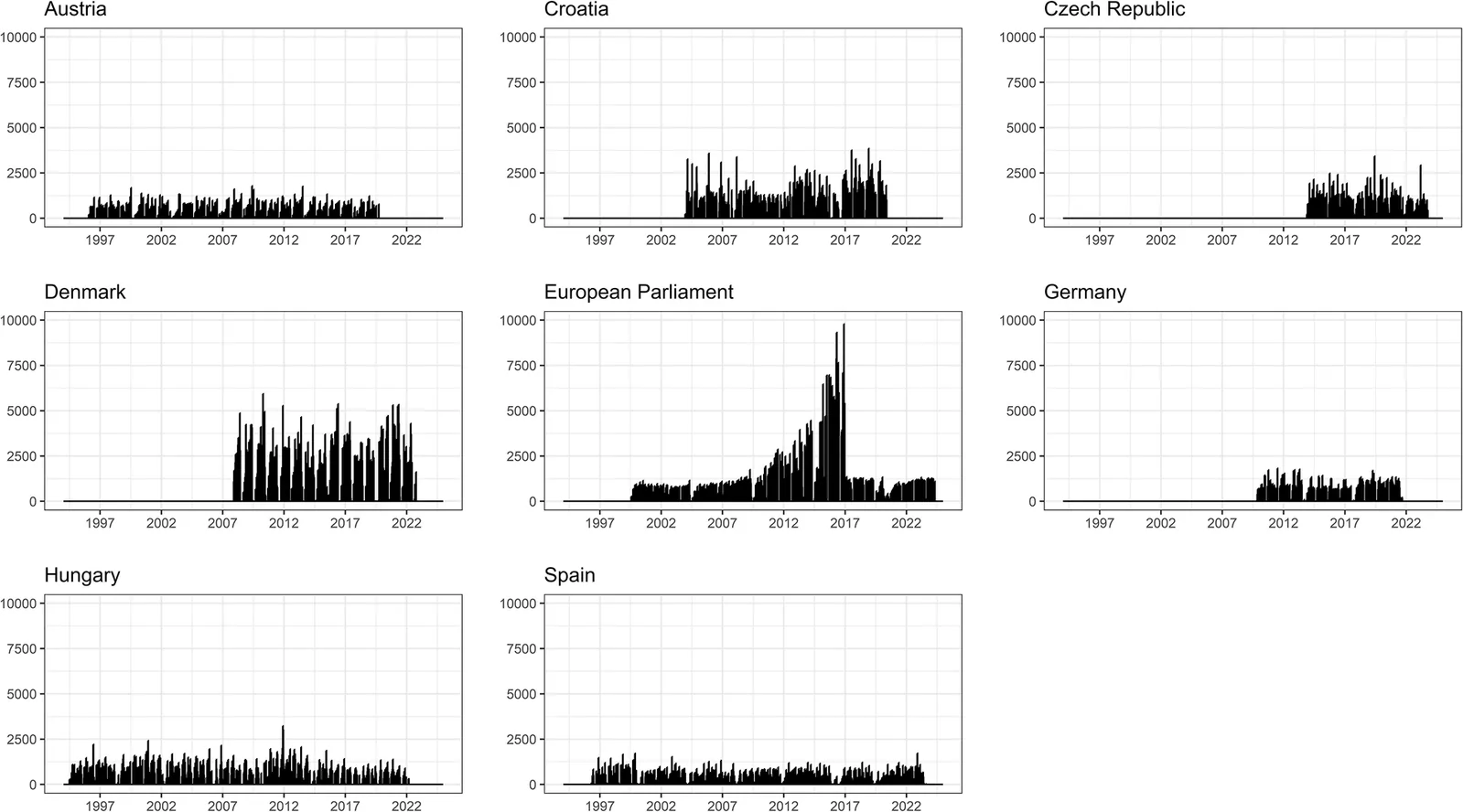
Speech Distribution.

Figure 1 provides an overview of the speech distribution in all datasets throughout the respective time frames. All data records show a relatively even distribution with partly repeating patterns over legislative periods, except for the EP. This difference can be explained by changes in the rules governing written contributions to debates^40^. These included the abolition of written declarations and a modification of the maximum number of questions for written answers in order to achieve greater efficiency^41^. If these are excluded using the written variable (see *Data Record*), the picture is comparable to that of the other datasets.

**Fig. 2:**
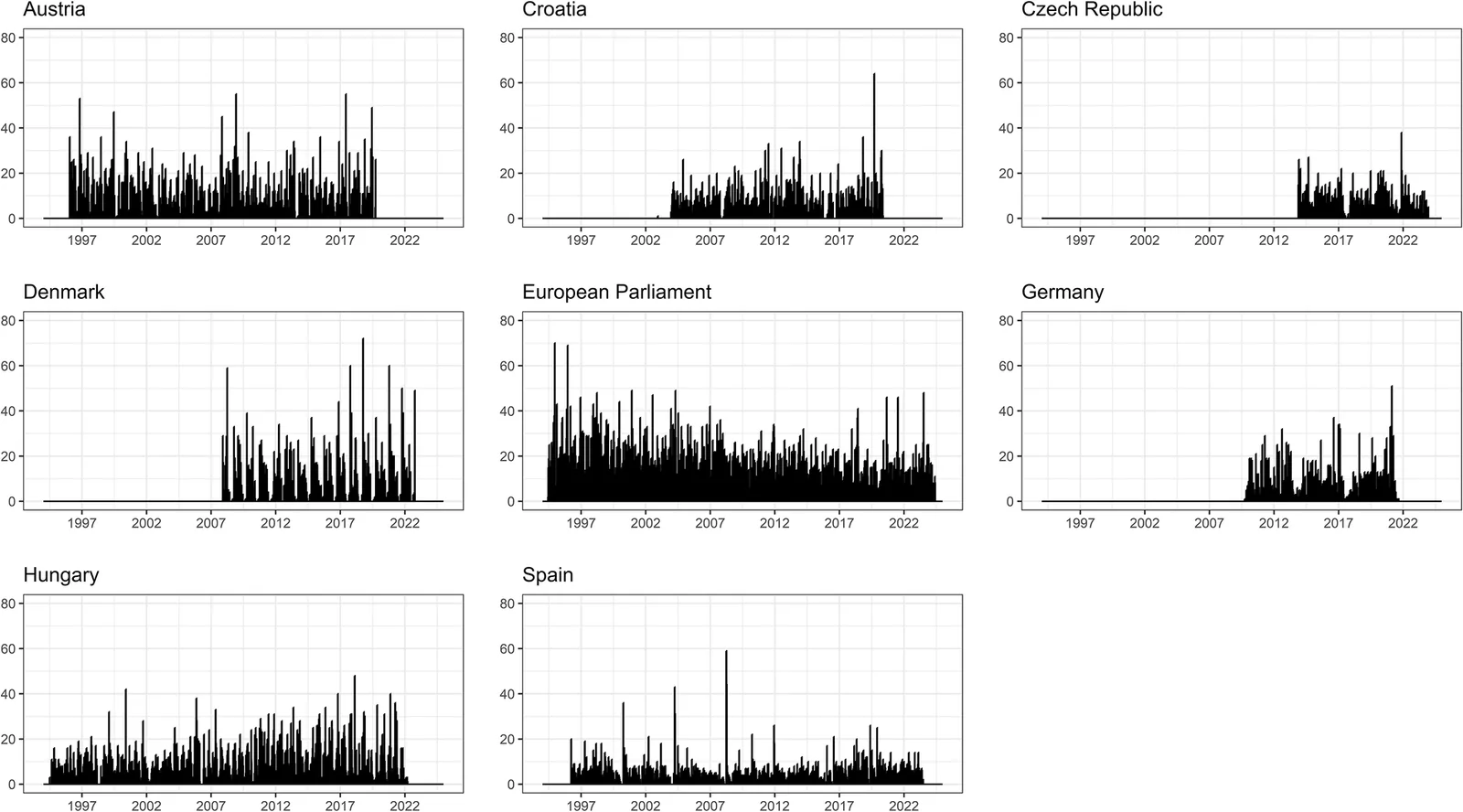
Bill Distribution.

**Fig. 3:**
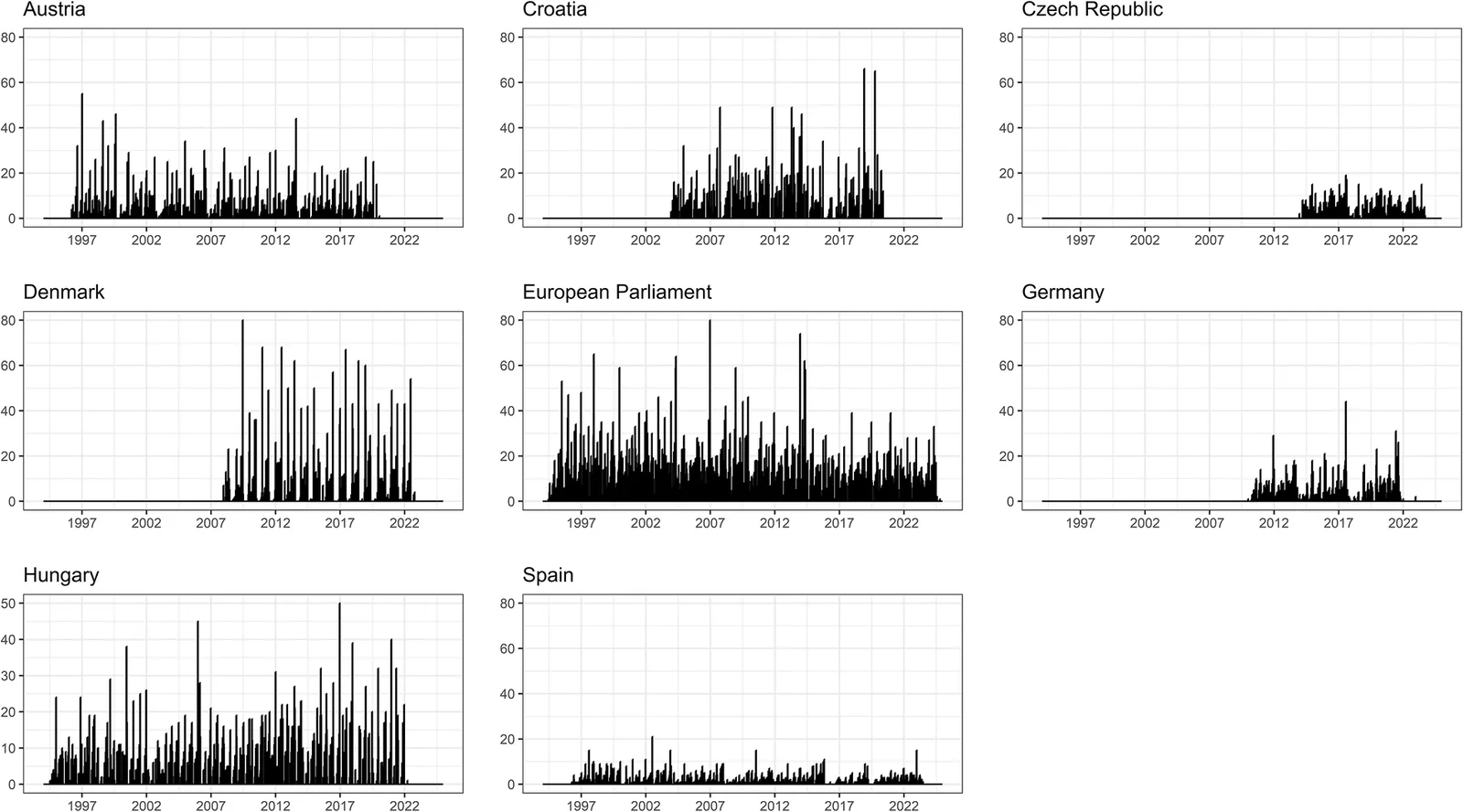
Law Distribution.

Figure 2 provides an overview of the bill distribution in all datasets throughout the respective time frames. Again, the datasets show a rather even distribution across the legislative periods with outliers, during which a particularly large number of bill proposals were introduced. Figure 3 provides an overview of the law distribution in all datasets throughout the respective time frames. Following the distribution of the bill datasets on average, the datasets show a rather even distribution across the legislative periods.

**Fig. 4:**
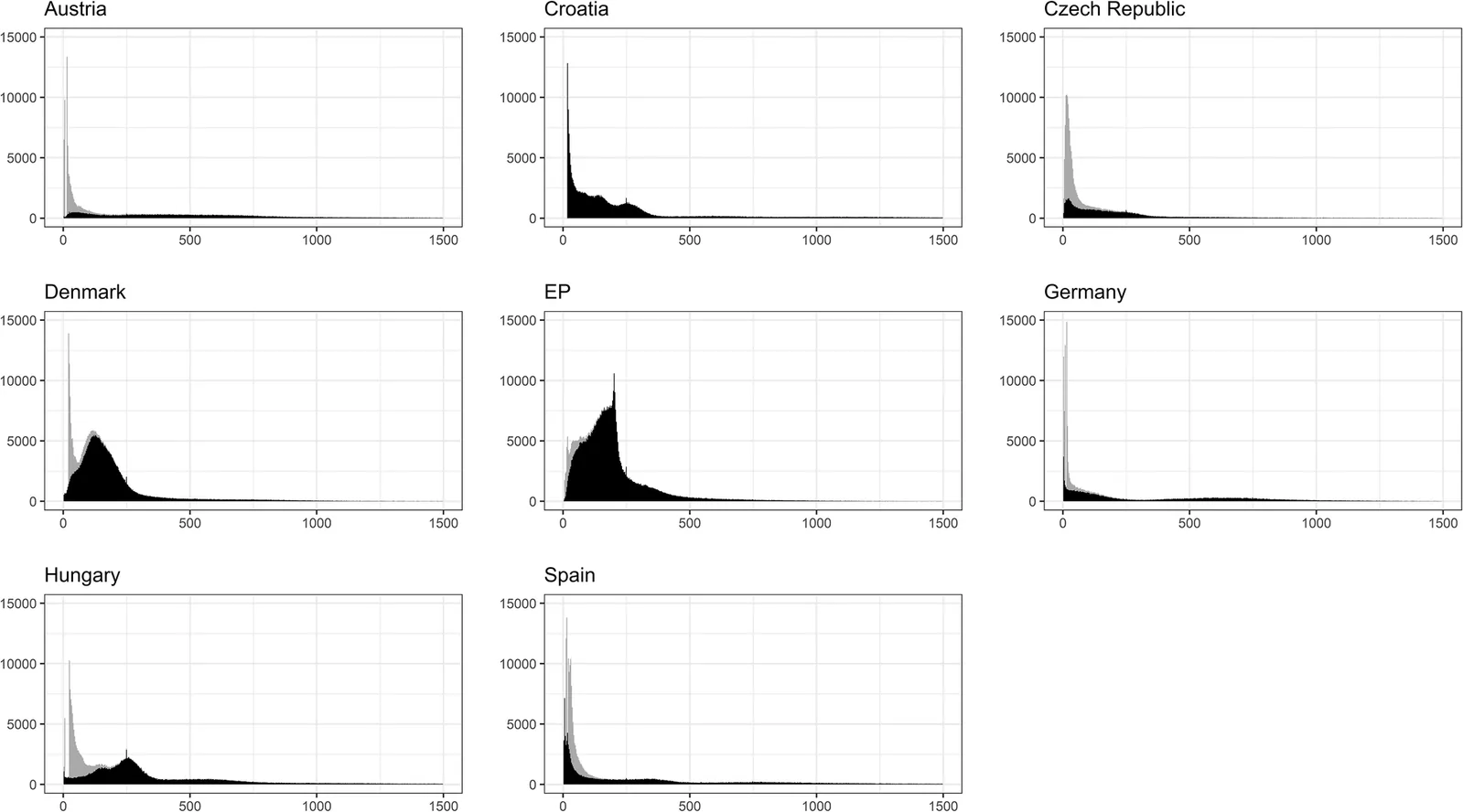
Speech Length Distribution.

Figure 4 displays an overview of the length of the speeches (number of words) per country. While the light grey distribution represents all speeches, the black distribution shows only those speeches that were not delivered by the chair. Two findings are worth highlighting here: First, in most parliaments, the chair’s speeches account for roughly half of all recorded speeches. Exceptions to this are Croatia, where the minutes generally do not include the chair’s statements, and the European Parliament, where these speeches account for a significantly smaller proportion in comparison to other parliaments. Second, when the chair’s speeches are included in the minutes, they are usually significantly shorter and more procedural in nature, with statements such as “The next speaker for Party XYZ is…” or “We now continue with agenda item XYZ”. As a result, the average number of words per speech across all parliaments ranges from 100 to 300 but rises to 200–500 without the chair’s speeches.

## Technical Validation

Validation was integral to the creation of the PLS dataset. Several R scripts were developed to facilitate the validation of critical aspects within the various corpora. The validation process was designed to ensure the completeness, consistency, and quality of the data both within and across corpora. In addition to the goal of implementing good scientific practice in the generation of datasets, a thorough validation process was necessary because the large number of different archives and data formats poses a variety of sources of error. The complete validation procedure involved both a mixture of human and automated validation as well as a mixture of structured and random validation. In the random selection procedure, we looked at randomly selected variable entries throughout the entire validation process to identify any overlooked inconsistencies and errors, which were then incorporated into the structured procedure. For each parliament, one round of validation was performed by a fluent speaker of the respective parliamentary language as well as a person that was not involved in the creation of the dataset before. This allowed us to rule out errors based on lacking language skills and, consequently, on misinterpreted structures. The structured procedure included five steps that we outline below in detail.

We started the validation by assessing the general completeness and checking for duplicates. To do so, we verified whether columns that should be unique (ID, text, link, etc.) contain no duplicates. Note that whether a column is expected to or should be unique strongly depends on the context. For instance, duplicate speech texts can be *true* duplicates (e.g., short responses such as “Thank you.”). Other cases (e.g., duplicate bill texts) required manual validation. These duplicate checks were followed by testing the completeness of the scraped corpus. Validating the performance of the scraper in terms of completeness was a delicate task, as we had to ensure that errors in the web scraper do not translate into our validation method. Therefore, ideally, the completeness of the scraped data was validated through both the original and alternative data sources or through alternative means. Alternatives were, for instance, different web sources (e.g., party websites) or different channels on the original source/parliamentary websites (e.g., filtering by MP or committee; search via the parliamentary “media library” instead of “data documentation”). After identifying the target number of observations through original and alternative data sources, we validated whether the number of observations in the scraped corpus matched the target number of observations overall and by groups. Thus, we confirmed whether all expected texts had been extracted from the source. This involved checking that no entries are missing. However, this step only confirmed the existence of text data; the completeness of the text content itself was addressed in Step 4.

In the second step, we focused on ensuring the accuracy and consistency of metadata across the dataset, including the standardization of text fields. We verified that categorical metadata (e.g., party names, speaker roles) is correctly labelled and consistent. For example, party names should match their official titles without variations or spelling errors. Furthermore, we checked that IDs (as retrieved from the data source) follow the expected format and structure specific to the source. Semi-automated tools such as regex can identify irregularities in structured fields like IDs, enabling efficient detection and correction of errors. Adjustments were applied iteratively to ensure consistency throughout the dataset.

Step three validated the cross-dataset linkage of the three datasets per country via the ID column, enabling further analyses. Validating these links was crucial to maintaining consistency across corpora and identifying any potential discrepancies. For the bill and law data, validation involved performing a full join between the two datasets. The number of observations in the joined dataset should match the total number of bills, indicating that each law is matched to a bill. It is important to note that the texts of bills and laws should not overlap, except in cases where both texts are extracted from the same document. When texts are extracted from the same document, their titles are expected to match.

For the relationship between bills and speeches, it is necessary to account for the fact that multiple bills can be associated with a single speech, and multiple speeches may reference the same bill. In the data, this relationship is managed by separating the IDs of multiple bills referenced in a single speech with commas (e.g., 19/50673/19, 19/4562/19). These entries were then unnested – converted from wide to long format – so that each bill ID has its own entry in the dataset. After unnesting, duplicate IDs were removed, ensuring that each bill ID appears only once in the speech-to-bill linking process. Following this preparation, the total number of bills was compared to the number of rows in the joined dataset. If the joined dataset contained more rows than the number of bills, this indicated the presence of IDs in the speech dataset that do not match any IDs in the bill dataset. Such discrepancies suggest errors in the IDs that required further investigation and correction. Some country datasets contain bills that are not debated in parliament and are therefore not associated with speeches. Some of these bills later become laws, which can be verified by linking the bill IDs to the law data.

To complement these structural checks, we additionally conducted a manual validation of the linkage quality. For each parliament, 50 bills were randomly sampled, irrespective of whether they are linked to speeches or laws. Independent research assistants who had not been involved in the data collection reconstructed the legislative procedure using only the bill title, initiation date, and link to the original parliamentary website. They recorded the proposal status, debate dates, and – where applicable – the adoption date and title of the corresponding law. These independently collected data were then compared with the automatically generated speech and law links in PLS. Across all sampled procedures, we found no discrepancies between the manually reconstructed procedures and the automatically generated links.

The first column of Table 12 shows how many speeches can be linked to a bill, the second column shows how many bills can be linked to laws, and the following columns—three and four—show these two ratios reversed. However, it is important to highlight that the raw number of links does reflect the quality of our ID variable as, for example, a link between a speech and a bill might not be present because the linkage procedure does not work or because there was no bill discussed in a given debate. Instead, the data offers insight into parliamentary traditions and procedures such as whether bills are debated in committees rather than on the floor or whether it is common for the opposition to draft bills that are very unlikely to pass. After the validation procedure, we found no remaining false positives or false negatives in the links between the three data types: speeches, bills, and laws.

**Table 12:** Linkage Ratios between Data Types.

Parliament	Speeches to Bills	Bills to Laws	Laws to Bills	Bills to Speeches
Austria	44.5%	51.2%	100%	83.4%
Croatia	57.7%	80.8%	100%	92.3%
Czech Republic	68.4%	39.7%	100%	68.2%
Denmark	33.9%	89.1%	100%	97.1%
European Parliament	34.0%	83.9%	100%	32.1%
Germany	23.6%	67.0%	100%	62.5%
Hungary	48.7%	57.4%	100%	76.3%
Spain	30.3%	37.3%	100%	58.3%

The fourth step ensures the quality and completeness of the text content (speeches, bills, laws) through an iterative process. This step involved extracting random samples, identifying and correcting errors, and testing for tokenization accuracy. These steps were repeated until no more error patterns were identified in a random sample of at least 10 texts. In the first step, a random sample of text data was extracted from the dataset, with each sampled row saved in its current processed and raw forms as local text files. The sampling process kept track of previously selected texts to ensure that no row is sampled more than once.

After sampling, errors in the text data were manually identified by language experts and corrected by the coder responsible for extraction and preprocessing. This process involved comparing processed texts, raw files, and original documents to ensure that the cleaned texts are accurate and complete representations of the originals. Key focus areas included encoding (ensuring characters are correctly encoded), cleaning (verifying that headers, footers, and other extraneous content have been removed), completeness (checking for completeness by reviewing patterns at the start and end of documents), targeted content (confirming that the text includes only the intended content; Errors, such as multiple target elements, e.g., two speeches in one, can be identified by skimming paragraphs in the sampled text), and hyphenation (ensuring hyphenated words have been correctly identified and de-hyphenated). Errors that stem from inaccuracies in the original documents (e.g., typos) were not corrected unless they introduced systematic bias. However, for errors introduced during preprocessing, adjustments were made to the corresponding code, typically in the script that processes raw texts into clean texts.

After each iteration, the current state of all sampled texts was saved, with filenames containing the row number and iteration number. This allowed for tracking progress and identifying whether new errors are introduced during subsequent corrections. To validate sentence tokenization, a sample of bills, laws, and speeches was selected. The number of sentences in the cleaned text was counted and compared to the count derived from “str_count”. Any discrepancies were used to identify lingering issues with tokenization.

As a final step, potential errors in the dataset were identified through outlier analyses and other exploratory validation techniques. This process involved creating descriptive visualizations and leveraging automated text analysis methods to detect anomalies that may indicate underlying issues. First, basic scatterplots and visualizations were used to identify inconsistencies in the dataset. These plots highlight relationships between key text features, and deviations from expected patterns can signal potential errors. Examples include: (1) Scatterplots of text length: This should show a linear correlation. Significant outliers may point to incorrectly processed or incomplete text. (2) Scatterplots of cleaned text length by raw text length: A linear relationship is expected; unusually short, cleaned texts may indicate issues during the cleaning process. (3) Scatterplots of text length by number of sentences: If sentence separation is possible, this plot can help evaluate the accuracy of tokenization and identify unusual cases. (4) Scatterplots of bill text length by law text length: A linear correlation is generally expected, with outliers potentially reflecting mismatched or incomplete data.

Second, we complemented the structured validation workflow with several exploratory corpus-level analyses designed to identify residual issues that might only become apparent during substantive data analysis. Specifically, we applied (1) sentiment analysis to identify unexpected sentiment patterns, (2) Wordfish to detect unusual lexical distributions, (3) unsupervised topic modelling to identify incoherent or irrelevant topics, and (4) TF-IDF comparisons across corpora to detect unexpectedly rare or missing tokens. These methods were not intended as standalone validation tools but rather as a final “stress test” of the corpus. Documents flagged as statistical outliers were manually inspected to assess whether they reflected extraction, preprocessing, or labelling errors. While a small number of outliers were identified, all represented substantively unusual parliamentary debates or legislative documents rather than errors in the corpus construction process, providing additional confidence in the quality of the final datasets.

Finally, regarding the add-on machine translation of the EP and HU speech corpora, no project-internal translation-quality assessment (e.g., back-translation, BLEU) was conducted. We instead relied on the established external validation literature benchmarking the Google Neural Machine Translation system against political science analyses of parliamentary text specifically: De Vries *et al*.^34^ demonstrated that Google Translate outputs preserve substantive meaning sufficiently for downstream political-text analyses (topic modeling, sentiment, keyword extraction) across multiple European languages, and Lucas *et al*.^35^ reported comparable performance for cross-lingual comparative work. Users requiring exact-wording fidelity (e.g., discourse or stylistic analyses) should consult these benchmarks, treat the readily-prepared translations with corresponding caution, or use the original-language material provided in the data for their own translations or task-specific validation exercises (e.g., back-translation on random samples, or human comparison on a stratified subsample) appropriate to their intended downstream analysis^36,37^.

## Usage Notes (Data Descriptors only, optional)

In addition to the freely accessible ParlLawSpeech data dumps, we have also set up a companion website that can be accessed at www.parllawspeech.org. This website is meant to give researchers the opportunity to provide feedback on the data and to feature all published work based on (any of) the PLS collection(s). Furthermore, the website provides a set of tutorials that offer users R code examples to get started quickly with using the data. These analytic examples also illustrate the analytic potential of the document linkage that PLS offers. For example, we show how to comparatively analyse the amount of debate across different bills, the varying partisan attention to specific bills, or the amount of legislative change that different bills experience in parliament before being adopted as binding law.

## Supplementary information

Supplementary Information

Supplementary Information

## Data Availability

ParlLawSpeech (DOI: https://doi.org/10.7802/2824) operates within an open science framework, with all data being openly published on a public repository. All datasets can be downloaded freely under the Open Access category from GESIS via https://search.gesis.org/research_data/SDN-10.7802-2824^38 ^.

## Code availability

In a supplementary document, we provide annotated code that we used as a template for validating our datasets. In addition, we provide documentation of R scripts implementing the machine-translation pipeline described above - sentence tokenization, local language detection, Google Cloud Translation API calls, and re-aggregation to speech level - as a separate file for inspection.

## References

[1] Proksch, S. & Slapin, J. *The Politics of Parliamentary Debate: Parties, Rebels and Representation*, 10.1017/cbo9781139680752 (2014).

[2] Peterson, A. & Spirling, A. Classification accuracy as a substantive quantity of interest: Measuring polarization in Westminster Systems. *Political Analysis* **26**, 120–128 10.1017/pan.2017.39 (2018).

[3] Rheault, L., Beelen, K., Cochrane, C. & Hirst, G. Measuring Emotion in Parliamentary Debates with Automated Textual Analysis. *PLoS ONE* **11**, e0168843 10.1371/journal.pone.0168843 (2016). 28006016 PMC5179059

[4] Valentim, V. & Widmann, T. Does Radical-Right Success Make the Political Debate More Negative? Evidence from Emotional Rhetoric in German State Parliaments. *Political Behavior* **45**, 243–264 (2021).

[5] Kostikova, A. *et al* Fine-Grained Detection of Solidarity for Women and Migrants in 155 Years of German Parliamentary Debates. *Proceedings of the 2024 Conference on Empirical Methods in Natural Language Processing* 5884–5907 10.18653/v1/2024.emnlp-main.337 (2024).

[6] Osnabrügge, M., Ash, E. & Morelli, M. Cross-Domain topic classification for political texts. *Political Analysis* **31**, 59–80 10.1017/pan.2021.37 (2021).

[7] Duthie, R., Budzynska, K. & Reed, C. Mining ethos in political debate. *Computational Models of Argument* **287**, 299–310 10.3233/978-1-61499-686-6-299 (2016).

[8] Thomas, M., Pang, B. & Lee, L. Get out the vote: Determining support or opposition from Congressional floor-debate transcripts. *Proceedings of EMNLP* **2006**, 327–335 (2006).

[9] Rheault, L. & Cochrane, C. Word embeddings for the analysis of ideological placement in parliamentary corpora. *Political Analysis* **28**, 112–133 10.1017/pan.2019.26 (2019).

[10] Kornilova & Eidelman, V. BillSum: A Corpus for Automatic Summarization of US Legislation. *Proceedings of the 2nd Workshop on New Frontiers in Summarization* 48–56 10.18653/v1/d19-5406 (2019).

[11] Rennard, V., Shang, G., Grari, D., hunter, J. & Vazirgiannis, M. FREDSum: A dialogue summarization corpus for French political debates. *Findings of the ACL*, 4241-4253 10.18653/v1/2023.findings-emnlp.280 (2023).

[12] Rodman, E. A Timely Intervention: Tracking the Changing Meanings of Political Concepts with Word Vectors. *Political Analysis* **28**, 87–111 10.1017/pan.2019.23 (2019).

[13] Erjavec, T. *et al*. The ParlaMint corpora of parliamentary proceedings. *Language Resources and Evaluation* **57**, 415–448 10.1007/s10579-021-09574-0 (2023). 35125984 PMC8807381

[14] Schwalbach, J. & Rauh, C. Collecting large-scale comparative text data on legislative debates. In *The Politics of Legislative Debate* (ed. Bäck, H., Debus, M. & Fernandes, J. M.) 91-109 10.1093/oso/9780198849063.003.0006 (Oxford Univ. Press, 2021).

[15] Sebők, M. *et al*. Comparative European legislative research in the age of large-scale computational text analysis: A review article. *International Political Science Review* **46**, 18–39 10.1177/01925121231199904 (2025).

[16] Katz, L. & Alexander, R. Digitization of the Australian Parliamentary Debates, 1998–2022. *Sci Data* **10**, 567 10.1038/s41597-023-02464-w (2023). 37633976 PMC10460412

[17] Fiva, J. H., Nedregård, O. & Øien, H. The Norwegian Parliamentary Debates Dataset. *Sci Data* **12**, 4 10.1038/s41597-024-04142-x (2025). 39747196 PMC11696194

[18] Simola, S., Nieminen, J. & Tukiainen, J. Finnish parliamentary speeches dataset. *Sci Data* **12**, 1063 10.1038/s41597-025-05056-y (2025). 40550807 PMC12185682

[19] Erjavec, T. *et al*. Multilingual comparable corpora of parliamentary debates ParlaMint 5.0, *CLARIN.SI* http://hdl.handle.net/11356/2004, (2025).

[20] Rauh, C. & Schwalbach, J. The ParlSpeech V2 data set: Full-text corpora of 6.3 million parliamentary speeches in the key legislative chambers of nine representative democracies. *Harvard Dataverse* 10.7910/DVN/L4OAKN (2020).

[21] Sylvester, C., Greene, Z. & Ebing, B. ParlEE plenary speeches data set: Annotated full-text of 21.6 million sentence-level plenary speeches of eight EU states *Harvard Dataverse*, 10.7910/DVN/ZY3RV7 (2022).

[22] Hunter, T. & Walter, S. International organizations in national parliamentary debates. *Rev Int Organ* **21**, 41–67 10.1007/s11558-024-09577-w (2026).

[23] Sebők, M., Molnár, C. & Takács, A. Levelling up quantitative legislative studies on Central-Eastern Europe: Introducing the ParlText CEE Database of Speeches, Bills, and Laws. *Intersections. East european Journal of Society and Politics* **10**, 106–125 10.17356/ieejsp.v10i4.1327 (2025).

[24] Kiss, R. & Sebők, M. Creating an Enhanced Infrastructure of Parliamentary Archives for Better Democratic Transparency and Legislative Research: Report on the OPTED forum in the European Parliament. *International Journal of Parliamentary Studies* **2**, 278–284 10.1163/26668912-bja10053 (2022).

[25] R Core Team. R. A Language and Environment for Statistical Computing. *R Foundation for Statistical Computing* https://www.R-project.org/ (2024).

[26] Harrison, J. Rselenium. R Bindings for ‘Selenium WebDriver’, version 1.7.9*. Comprehensive R Archive Network* https://cran.r-project.org/web/packages/RSelenium/index.html, (2022).

[27] Temple Lang, D. XML: Tools for Parsing and Generating XML Within R and S-Plus. version 3.99-0.16.1. *Comprehensive R Archive Network*, 10.32614/CRAN.package.XML (2024).

[28] Wickham, H. rvest: Easily Harvest (Scrape) Web Pages. version 1.0.4. *Comprehensive R Archive Network* 10.32614/CRAN.package.rvest (2024).

[29] Schwalbach, J. Mind the context! The role of theoretical concepts for analyzing legislative text data. *Research & Politics* **11** 10.1177/20531680241277466 (2024).

[30] Wickham, H. stringr: Simple, Consistent Wrappers for Common String Operations. version 1.5.1. *Comprehensive R Archive Network*, 10.32614/CRAN.package.stringr (2023).

[31] Gagolewski, M. stringi. Fast and Portable Character String Processing in R. *Journal of Statistical Software* **103**, 1–59 10.18637/jss.v103.i02 (2022).

[32] Hester, J. & Bryan, J. glue. Interpreted String Literals, version 1.8.0. *Comprehensive R Archive Network* https://CRAN.R-project.org/package=glue, (2024).

[33] Ooms, K. pdftools. Text Extraction, Rendering and Converting of PDF Documents, version 3.4.0. *Comprehensive R Archive Network* https://CRAN.R-project.org/package=pdftools, (2023).

[34] De Vries, E., Schoonvelde, M. & Schumacher, G. No Longer Lost in Translation: Evidence that Google Translate Works for Comparative Bag-of-Words Text Applications. *Political Analysis* **26**, 417–430 10.1017/pan.2018.26 (2018).

[35] Lucas, C. *et al*. Computer-Assisted text analysis for comparative politics. *Political Analysis* **23**, 254–277 10.1093/pan/mpu019 (2015).

[36] Licht, H. & Lind, F. Going cross-lingual: A guide to multilingual text analysis. *Computational Communication Research* **5**, 1–31 10.5117/CCR2023.2.2.LICH (2023).

[37] Lind, F. *et al*. Grounding the Comparative Turn in Communications: A Framework for Validating Multilingual Computational Text Analysis. *Computational Communication Research* **7**, 1.26 10.5117/CCR2025.1.13.LIND (2025).

[38] Schwalbach, J., Hetzer, L., Proksch, S. O., Rauh, C. & Sebők, M. ParlLawSpeech. *GESIS*10.7802/2824 (2025).42763328

[39] Döring, H. & Regel, S. Party Facts: A database of political parties worldwide. *Party Politics* **25**, 97–109 10.1177/1354068818820671 (2019).

[40] Sorace, M. European Parliament Debating in a Legislature with Competing Incentives. In *The Politics of Legislative Debate* (ed. Bäck, H., Debus, M. & Fernandes, J. M.) 304-328 10.1093/oso/9780198849063.003.0016 (Oxford Univ. Press, 2021).

[41] Kotanidis, S. *General revision of the European Parliament’s Rules of Procedure: Achieving greater transparency and efficiency as of January 2017* 10.2861/338993 (European Union, 2017).

