# Supplementary material for: The ParlLawSpeech Parliamentary Text Data Collection > 3 Million Parliamentary Bills, Laws, and Speeches from 8 Legislatures
Source: Sci Data. 2026 Sep 19;13:1327. doi: 10.1038/s41597-026-08335-4 (PMC13589736; doi:10.1038/s41597-026-08335-4)
Supplement: Supplementary file 1 [file 41597_2026_8335_MOESM1_ESM.pdf]

Validation Workflow – *ParlLawSpeech*

Overview

This file documents the validation workflow applied to the ParlLawSpeech dataset. The purpose of the validation process is to ensure the internal consistency, completeness, and structural integrity of the collected data.

More specifically, the validation procedures are designed to ensure that:

- Observations are unique and identifiers are consistent.
- Missing data and empty text fields are systematically identified.
- Scraped documents correspond to official legislative records.
- Links between speeches, bills, and laws are correctly captured.
- Metadata fields follow consistent formatting rules.
- Text content is clean and accurately reflects the original documents.
- Potential outliers or anomalies in legislative text length are identified and inspected.

The workflow consists of six main stages:

1. **General completeness and duplicate checks**
   - 1.1. Speeches
   - 1.2. Bills
   - 1.3. Laws
2. **Validation of scraped data coverage**
   - 2.1. Speeches
   - 2.2. Bills
   - 2.3. Laws
3. **Links between the corpora**

3.1. Links between bills and laws

3.2. Links between bills, laws, and speeches

4. **Validation of metadata variables**

   4.1. Speeches: party, agenda, date, speaker, chair, and period variables

   4.2. Bills: initiator, committee, status, and period variables

   4.3. Laws: period and adoption date variables

5. **Manual inspection of text content**

   5.1. Speeches

   5.2. Bills

   5.3. Laws

6. **(Exploratory) Outlier detection**

While the steps described below reflect the general validation workflow applied across the dataset, not all procedures were required for every country. Some checks were only relevant in specific cases depending on the structure and availability of source data, and additional validation steps were performed where necessary. The workflow presented here therefore summarizes the standard validation procedures implemented across the project.

The validation was implemented using `R`. Examples of code snippets are provided in this document.

0 Setup and Data Loading

The validation scripts rely primarily on the `tidyverse` ecosystem and several supporting packages for text processing and visualization. A fixed random seed is defined to ensure reproducibility of all random sampling procedures used throughout the validation workflow.

```
# Clear memory
rm(list = ls())
options(scipen = 999)

# Load packages
library(tidyverse)
library(lubridate)
library(quanteda)
library(ggplot2)
library(writexl)
library(stringr)
library(ggpubr)
```

```r

# Set directory paths
data_path <- "PATH_TO_DATA/"
val_path <- "PATH_TO_VALIDATION_OUTPUT/"

# Load datasets
speeches <- readRDS(paste0(data_path, "Corpus_speeches.RDS"))
laws <- readRDS(paste0(data_path, "Corpus_laws.RDS"))
bills <- readRDS(paste0(data_path, "Corpus_bills.RDS"))

# Set random seed for reproducible validation samples
set.seed(01011999)
```

1 General Completeness and Duplicate Checks

The first stage examines whether observations are unique and whether key variables contain missing values.

1.1 Speeches

Uniqueness checks are performed for the entire dataset as well as for key identifier variables.

```r
# Check uniqueness of rows
nrow(speeches) == nrow(unique(speeches))

# Check duplicates in key variables
length(speeches$speech_ID) -
  length(unique(speeches$speech_ID))

length(speeches$procedure_ID) -
  length(unique(speeches$procedure_ID))

length(speeches$text) -
  length(unique(speeches$text))
```

Missing text values and empty strings are also examined.

```r
unique(is.na(speeches$text))
which(speeches$text == "")
min(nchar(speeches$text))
```

1.2 Bills

Similar checks are conducted for the legislative proposals dataset.

```
# Check uniqueness
nrow(bills) == nrow(unique(bills))

# Check duplicates
length(bills$bill_text) -
  length(unique(bills$bill_text))

# Missing text
length(which(is.na(bills$bill_text)))
length(which(bills$bill_text == ""))
```

1.3 Laws

For adopted legislation, additional checks ensure that identifiers and texts are unique.

```
nrow(laws) == nrow(unique(laws))

length(laws$procedure_ID) ==
  length(unique(laws$procedure_ID))

length(laws$law_ID) ==
  length(unique(laws$law_ID))
```

2 Validation of Scraped Data Coverage

To ensure that all relevant legislative documents have been collected, counts of speeches, bills, and laws in the dataset are compared with official sources as well as alternative sources, where available.

Listed below are possible variations of filtering mechanisms applied to verify observation counts across different groupings.

2.1 Speeches

```
speeches_party_period <- speeches %>%
  group_by(party, period) %>%
  summarize(number_of_speeches_data = n()) %>%
  ungroup()
```

The resulting counts are manually compared to the number of speeches that can be identified through alternative sources such as party websites or filtering options on official parliamentary databases.

2.2 Bills

```
bills_periods <- bills %>%
  group_by(period) %>%
  summarize(number_of_bills_data = n()) %>%
  ungroup()
```

The resulting counts are manually compared to the number of bills listed on the official parliamentary website or alternative sources for each legislative period.

2.3 Laws

```
laws_year <- laws %>%
  mutate(year = year(adoption_date)) %>%
  filter(year >= START_YEAR & year <= END_YEAR) %>%
  group_by(year) %>%
  summarize(number_of_laws_data = n()) %>%
  ungroup()
```

The resulting counts are compared with the number of laws listed in official legislative databases after filtering the database for the same year range.

3 Links Between the Corpora

Next, links between speeches, bills, and laws are verified.

3.1 Links Between Bills and Laws

Bills and laws are linked through shared legislative procedure identifiers. The relationship is not necessarily one-to-one, as a single bill may result in multiple legal acts and vice versa. Country-specific exceptions and differences in the temporal coverage of the corpora are also taken into account when evaluating unmatched observations.

To validate the linkage, the bill and law corpora are joined using the common procedure identifier. Where multiple procedure identifiers are stored in one observation, the identifiers are first separated into individual rows and whitespace is removed. A full join retains all observations from both corpora and therefore allows bills without an adopted law and laws without a corresponding bill record to be identified.

```
bill_law_links <- bills %>%
  select(procedure_ID,
         title_bill,
         status,
         initiation_date,
         bill_text) %>%
```

```
  separate_rows(procedure_ID, sep = ",") %>%
  mutate(procedure_ID = str_trim(procedure_ID)) %>%
  full_join(
    laws %>%
      select(procedure_ID,
             title_law,
             adoption_date,
             law_text) %>%
      separate_rows(procedure_ID, sep = ",") %>%
      mutate(procedure_ID = str_trim(procedure_ID)),
    by = "procedure_ID"
  )
```

Laws without a corresponding bill record are identified through missing bill metadata in the joined dataset. These observations are manually inspected to determine whether they reflect linkage errors, country-specific exceptions, or differences in the temporal coverage of the corpora.

```
unmatched_laws <- bill_law_links %>%
  filter(is.na(title_bill),
         !is.na(title_law))
```

Bills without corresponding law records are not generally treated as linkage errors, since legislative proposals may be rejected, withdrawn, or remain unfinished at the end of the observation period. However, bills with a legislative status indicating adoption are manually inspected to verify that a corresponding law has been correctly linked. As status labels differ across parliaments, the relevant values are defined separately for each corpus.

```
unmatched_bills <- bill_law_links %>%
  filter(!is.na(title_bill),
         is.na(title_law))

adopted_without_law <- unmatched_bills %>%
  filter(status %in% adopted_status)
```

3.2 Links Between Bills, Laws and Speeches

The linkage between speeches and legislative documents is validated by checking whether procedure identifiers referenced in speeches occur in the bill and law datasets.

As with bills and laws, these relationships are not necessarily one-to-one. A single speech may refer to multiple legislative procedures, while multiple speeches usually refer to the same legislative procedure. The specific structure of these relationships depends on the institutional context and parliamentary practices in each country.

Where multiple procedure identifiers are stored in one speech observation, the identifiers are first separated into individual rows and whitespace is removed. The resulting speech links are then

aggregated by legislative procedure. This retains both the number and dates of the parliamentary debates associated with each procedure.

```
speech_link_summary <- speeches %>%
  filter(!is.na(procedure_ID),
         procedure_ID != "") %>%
  select(procedure_ID, date) %>%
  separate_rows(procedure_ID, sep = ",") %>%
  mutate(procedure_ID = str_trim(procedure_ID)) %>%
  distinct(procedure_ID, date) %>%
  group_by(procedure_ID) %>%
  summarize(
    number_of_debate_dates = n_distinct(date),
    debate_dates = paste(
      sort(unique(date)),
      collapse = "; "
    ),
    .groups = "drop"
  )

bill_speech_links <- bills %>%
  select(procedure_ID,
         title_bill,
         initiation_date,
         status) %>%
  separate_rows(procedure_ID, sep = ",") %>%
  mutate(procedure_ID = str_trim(procedure_ID)) %>%
  full_join(
    speech_link_summary,
    by = "procedure_ID"
  )
```

Speech identifiers without a corresponding entry in the bill corpus are identified through missing bill metadata in the joined data. These observations are manually inspected to determine whether they reflect incorrectly extracted identifiers, formatting inconsistencies, or country-specific exceptions.

```
unmatched_speech_ids <- bill_speech_links %>%
  filter(is.na(title_bill)) %>%
  select(procedure_ID,
         number_of_debate_dates,
         debate_dates)
```

Any unmatched identifiers are manually investigated with reference to the original parliamentary sources. Institutional exceptions and differences in the temporal coverage of the corpora are taken into account when evaluating these cases.

3.3 Human Validation of Linkage Quality

To complement the structural linkage checks described above, an independent manual validation of the linkage quality is performed.

For each parliament, a simple random sample of 50 legislative procedures is drawn from the bills corpus, irrespective of whether the procedures are linked to speeches or laws in the final dataset.

```
link_validation_sample <- bills %>%
  select(procedure_ID,
         title_bill,
         initiation_date,
         bill_link) %>%
  slice_sample(n = 50) %>%
  mutate(
    status_manual = NA_character_,
    number_of_debate_dates_manual = NA_integer_,
    debate_dates_manual = NA_character_,
    adoption_date_manual = as.Date(NA),
    law_title_manual = NA_character_
  )

write_xlsx(
  link_validation_sample,
  paste0(val_path,
         "manual_link_validation_sample.xlsx")
)
```

The sampled procedures are provided to independent coders who have not been involved in the development of the extraction and linkage pipeline. Only the information required to locate the procedure in the official parliamentary sources is provided. The speech and law links contained in PLS are not provided to the coders.

Using the official parliamentary sources, the coders independently record the proposal status, whether and when the proposal was debated in parliament, and, where applicable, the adoption date and title of the corresponding law.

After coding, the annotated table is joined to the linkage datasets created above. The bill–speech linkage supplies the automatically derived debate dates and their number, while the bill–law linkage supplies the status and metadata of the corresponding law.

```
manual_validation <- read_xlsx(
  paste0(val_path,
         "manual_link_validation_completed.xlsx")
)

manual_link_comparison <- manual_validation %>%
  left_join(
```

```
    bill_speech_links %>%
      select(procedure_ID,
             status,
             date),
    by = "procedure_ID"
  ) %>%
  left_join(
    bill_law_links %>%
      select(procedure_ID,
             adoption_date,
             title_law) %>%
      distinct(),
    by = "procedure_ID"
  )
```

Potential discrepancies between the independently collected information and the PLS metadata are then identified manually. All potential discrepancies are manually inspected against the original parliamentary sources. If systematic errors are identified, the corresponding extraction or linkage scripts are revised and the validation process is repeated.

3.4 Human Validation of Linkage Quality

To complement the structural linkage checks described above, an independent manual validation of the linkage quality is performed.

For each parliament, a simple random sample of 50 legislative procedures is drawn from the bills corpus, irrespective of whether the procedures are linked to speeches or laws in the final dataset. The variables included in the sample differ slightly across parliaments depending on the information available from the respective official sources.

```
link_validation_sample <- bills %>%
  select(title_bill,
         initiation_date,
         bill_link,
         procedure_ID) %>%
  slice_sample(n = 50) %>%
  mutate(
    status_manual = NA_character_,
    debate_date_manual = NA_character_,
    adoption_date_manual = NA_character_,
    law_title_manual = NA_character_)

write_xlsx(
  link_validation_sample,
  paste0(val_path, "manual_link_validation_sample.xlsx")
)
```

The sampled procedures are provided to independent coders who have not been involved in the development of the extraction and linkage pipeline. Only the information required to locate the procedure in the official parliamentary sources is provided. The automatically generated speech and law links contained in PLS are not provided to the coders.

Using the official parliamentary sources, the coders independently reconstruct each legislative procedure and record the proposal status, whether and when the proposal was debated in parliament, and, where applicable, the adoption date and title of the corresponding law.

After coding, the manually collected information is compared with the corresponding bill, speech, and law records in PLS. Bill–speech and bill–law links are assessed separately, following the linkage structure described in Sections 3.1 and 3.2. Any discrepancies are manually inspected to determine whether they reflect errors in the extraction or linkage procedures. If systematic errors are identified, the corresponding extraction scripts are revised and the validation process is repeated.

4 Manual Inspection of Text Content

To identify potential scraping errors or truncated texts, a stratified sample of documents is drawn from each parliamentary period. Extreme cases based on text length are also included in the sample.

```
speeches_stratified <- speeches %>%
  group_by(period) %>%
  sample_n(10) %>%
  mutate(word_count = str_count(text, " "))
```

The sampled texts are exported for manual inspection.

```
write_xlsx(speeches_stratified,
           paste0(val_path, "speeches_stratified.xlsx"))
```

Equivalent procedures are applied to the bill and law corpora.

This validation step is conducted as an iterative process. Random text samples are repeatedly extracted from the corpora and manually inspected to identify recurring error patterns, such as incorrectly encoded characters, remaining headers or footers, truncated texts, or cases where multiple target texts were incorrectly merged into a single observation. Identified error patterns are subsequently addressed through programmatic adjustments to the text-cleaning procedures and the process is repeated until no further systematic errors are observed in random samples.

As part of this step, sentence segmentation is also tested to ensure that the cleaned texts can be reliably tokenized for downstream text analysis. This involves comparing sentence counts obtained through regular expression-based heuristics with those produced by sentence tokenization tools

(e.g., quanteda). Large discrepancies are manually inspected to detect remaining formatting or cleaning issues.

5 (Exploratory) Outlier Detection

Finally, potential anomalies in the textual data are examined using simple descriptive diagnostics. These diagnostics help identify cases where the scraped or processed texts may contain remaining errors, such as truncated texts, duplicated content, or incomplete cleaning.

First, differences between bill texts and the corresponding adopted laws are examined. Large discrepancies in text length may indicate issues such as incomplete scraping or incorrect document matching.

```
billXlaw <- select(bills, procedure_ID, bill_text) %>%
  right_join(select(laws,
                  procedure_ID,
                  law_text),
            by = "procedure_ID")

billXlaw$char_law <- nchar(billXlaw$law_text)
billXlaw$char_bill <- nchar(billXlaw$bill_text)

billXlaw$char_diff <- sqrt(
  (billXlaw$char_law - billXlaw$char_bill)^2
)
```

Large discrepancies between text lengths are flagged and inspected manually.

Additional descriptive diagnostics are used to detect unusual text patterns. For example, text length is compared with the number of punctuation characters. As punctuation generally scales with text length, strong deviations from this relationship may indicate remaining formatting errors or truncated texts.

```
bills$num_words <- str_count(bills$bill_text, "\\S+")
bills$num_punctuation <- str_count(bills$bill_text, "[[:punct:]]")

ggplot(bills, aes(x = num_punctuation, y = num_words)) +
  geom_point() +
  geom_smooth(method = "lm", se = FALSE) +
  labs(
    x = "Number of Punctuation Characters",
    y = "Number of Words",
    title = "Text Length vs Punctuation"
  ) +
  theme_minimal()
```

Another diagnostic compares the length of cleaned texts with the corresponding raw texts. Large deviations between raw and cleaned text length may indicate that important text sections were removed during the cleaning process.

```
num_raw_words <- sapply(strsplit(bills$text, "\\s+"), length)
num_cleaned_words <- sapply(strsplit(bills$bill_text, "\\s+"), length)

word_count_data <- data.frame(
  num_raw_words = num_raw_words,
  num_cleaned_words = num_cleaned_words
)

ggplot(word_count_data, aes(x = num_raw_words, y = num_cleaned_words)) +
  geom_point() +
  geom_smooth(method = "lm", se = FALSE) +
  labs(
    x = "Number of Words (Raw Text)",
    y = "Number of Words (Cleaned Text)",
    title = "Cleaned vs Raw Text Length"
  ) +
  theme_minimal()
```

An additional diagnostic compares text length with the number of sentences to verify that sentence boundaries are detected correctly.

```
bills$num_sentences <- str_count(bills$bill_text, "[.!?]")

ggplot(bills, aes(x = num_sentences, y = num_words)) +
  geom_point() +
  geom_smooth(method = "lm", se = FALSE) +
  labs(
    x = "Number of Sentences",
    y = "Number of Words",
    title = "Text Length vs Sentence Count"
  ) +
  theme_minimal()
```

Observations that strongly deviate from these expected relationships are manually inspected to determine whether they reflect genuine document characteristics or remaining data-processing errors.
