# Supplementary material for: The ParlLawSpeech Parliamentary Text Data Collection > 3 Million Parliamentary Bills, Laws, and Speeches from 8 Legislatures
Source: Sci Data. 2026 Sep 19;13:1327. doi: 10.1038/s41597-026-08335-4 (PMC13589736; doi:10.1038/s41597-026-08335-4)
Supplement: Supplementary file 2 [file 41597_2026_8335_MOESM2_ESM.html]

ParlLawSpeech documentation


## Table of contents

- Context and motivation
- How and what to translate?
- European Parliament
  - Sentence tokenization
  - Local language detection
  - Machine translation
  - Re-aggregation to speech level
  - Result
- Hungarian parliament (Országgyűlés)
  - Machine translation
  - Result
- API usage and budget consumption
- Data and further documentation

# ParlLawSpeech documentation

Machine translation of parliamentary speeches

Author

Christian Rauh

Modified

September 29, 2023


## Context and motivation

The ***OPTED initiative*** (European Union Horizon 2020 research & innovation programme / Grant Agreement no. 951832) is a design study that aims to show that and how an infrastructure for large-scale political text analysis could enhance systematic insights into the functioning of European democracies.

In this context, the team of ***OPTED Work Package 5*** has created ***ParlLawSpeech***, an encompassing new corpus offering readily machine-readable texts of the debates, bills, and laws from seven European parliaments. Large-scale comparative analysis of such text data is, however, significantly constrained by *language barriers* (see also OPTED Work Package 6)***.*** Our design study thus also included some budget for inquiring *the potential of machine translation for political text data*.

We accordingly decided to use this budget for *complementing some of our original-language parliamentary text corpora with machine translations in the English language.* This way, ParlLawSpeech provides additional data that can be used by a alrger number of scholars for either (a) validation studies regarding machine translation or (b) substantial analyses of parliamentary democracies for which language barriers have persisted thus far.

To ensure that these additional data and their generation are transparent for prospective users and can be reproduced in other validation studies, *we document our respective choices and the implementation of our machine translations here*.

## How and what to translate?

In terms of machine translation tools, we decided to use ***Google’s Neural Machine Translation*** system. While more and more machine translation models become publicly available (e.g., here) this choice is driven by three considerations:

- There are already *validation studies of Google Translate* for political text analysis, using a.o. parliamentary speeches (esp. De Vries et al. 2018 or Lucas et al., 2015). This provides relevant benchmarks for prospective users of our translated data while it also facilitates using them in cumulative validation efforts by the text analysis community.
- Compared to other tools, Google Translate still offers *the largest number of language pairs* available for translation, which is particularly relevant for multilingual parliaments such as the European Parliament (see below).
- The Google Translate model can be accessed on powerful servers through a *dedicated API* which enables us to handle large amounts of text data without additional investments in local computational hardware.

However, using this service at scale is *subject to fees* – in September 2023 amounting to 20$ per 1 million translated text characters (where automatic, server-side language detection is included). Given a limited budget, we thus had to make *further choices* regarding which elements of ParlLawSpeech to translate.

In this regard we first decided to ***focus on*** ***parliamentary debates and the speeches***therein, rather than on the bill or law texts in our corpora: large amounts of speech over time and parliaments offer insigths on the widest number of substantial research questions that could be or partially have been addressed in the political science literature.

But even with this restriction, the amount of text in our data by far exceeds the available translation budget - considering only speech corpora in ParlLawSpeech already amounts to more than three billion characters and corresponding translation costs of more than 60,600$ of translation costs at current Google prices.

Weighing respective cost estimations for our individual parliamentary speech corpora against potential benefits for the wider political science community, we decided to translate the debates of the ***European Parliament*** **(EP)** as well as of the ***Hungarian parliament (Országgyűlés)*** - together accounting for around ***25,000$ of translation costs*** (see also below).

We consider the EP as particualrly important for translation as it is the only true supranational but also decidedly multilingual parliament (which stopped providing official translations around November 2012). And we consider the Hungarian Parliament as particularly relevant for translation as it operates in a country for which many analysts attest a declining quality of democracy while it uses a language that is much more rarely spoken or read than other (western) European languages.

Especially for these two parliaments, thus, ***access to all-English text corpora of speeches*** seems to be most valuable for political science. In the remainder we document how the respective and initially validated text corpora in the ParlLawSpeech collection have been machine translated.

## European Parliament

For the European Parliament (EP), the original ParlLawSpeech collection starts in July 1999 and ends in April 2019, containing more than *500.000 indivdial speeches*.

It initially contains the *official translations* that the EP itself has published in its archives up until November 2012. But already in this early period, our local language detection tools (using the small fastText model as well as the compact language detectors in versions 2 and 3) suggest that many speeches have been *wrongly classified* by the EP as English.

After November 2012, speeches are *only represented in the language that the respective speaker has chosen*. A particular challenge here is that many speakers actually *switch languages* when giving a speech to the European parliament - our initial analyses suggest that this is true for at least 6% of all speeches.

#### Sentence tokenization

Because of these language switches and based on multiple prior analyses and tests (a.o. here), we decided to *operate on the sentence- rather than on the speech level* for this particular corpus.

That is, we first split each EP speech into individual sentences (using the tidytext tokenizers that showed better results that those offered by spacyR in qualitative analyses) along the code below.

Code

```
library(tidyverse)       # CRAN v2.0.0
library(tidytext)        # CRAN v0.4.1
library(progress)        # CRAN v1.2.2

# EP speech corpus ####
# Validated PLS Version including a unique row ID
speeches <- read_rds("./data/EP_speeches_PLS-original.rds") %>% 
  select(c(id, text))

# Sentence tokenization - full corpus ####
# with tidytext
start <- Sys.time()
sentences <- speeches %>%
    group_by(id) %>%
    unnest_tokens(output = sentence,
                  input = text,
                  token = "sentences",
                  to_lower = F) %>%
    ungroup()
duration <- Sys.time() - start
duration # 52 secs on Dell

# Export
write_rds(sentences, "./data/EP_speeches_PLS-SentenceTokenized.rds")
```

Then we carefully inspected the sentence tokenization output along the following code:

Code

```
sent <- read_rds("./data/EP_speeches_PLS-SentenceTokenized.rds")


# Inspect ####

# Some sentence parameters
sent$wordcount <- str_count(sent$sentence, "\\w+")
sent$chars <- nchar(sent$sentence)
sent$letters <- str_count(sent$sentence, "[A-Za-z]")

# Number of detected sentences by speech (id)
spe.sent <- sent %>% 
  group_by(id) %>% 
  summarise(sentences = n())

# Wordcount inspection
summary(sent$wordcount)
test <- sent %>% filter(wordcount > 32) # 3rd Quartile - lots of normal stuff
test <- sent %>% filter(wordcount > 100) # Much looks correct, lots on non-english on first impression  - sometimes agenda items with doc titles and sponsor etc show up here
test <- sent %>% filter(wordcount > 1000) # 12, all agenda items (probably not spoken words but no language errors)
test <- sent %>% filter(wordcount < 14) # 1st Quartile - normal short sentences
test <- sent %>% filter(wordcount < 7) # 230k still - reasonable stuff, lots of bracketed interventions
test <- sent %>% filter(wordcount == 0) # 616 - Only puntuation and formating markers like ⁂ - probably need to be excluded from  language detection and translation

# Character count
summary(sent$chars)
test <- sent %>% filter(chars < 3) # 2073 - ordered or decimal numbers (like "1."), abbreviations / initials (like "M."), and individual punctuation characters
# Her slight issues may occur when aggregating this pack to the speech level, e.g. where the tokenizer has split a decimal number - maybe manually correct after aggregation?

# Letter count
summary(sent$letters)
test <- sent %>% filter(letters <= 3) # 7850 - lots of greek and cyrillic (well...) - these should not be excluded from language detection and translation!

# str_count("Κυρία Πρόεδρε, κάθε χώρα της Ευρωπαϊκής Ένωσης", "\\w") # counts this as letters
sent$letters <- str_count(sent$sentence, "\\w") - str_count(sent$sentence, "[0-9]") - str_count(sent$sentence, fixed("_")) # All letters (from whatever alphabet)

summary(sent$letters)
test <- sent %>% filter(letters <= 3) # 5241 - lots of valid examples ("How?")
test <- sent %>% filter(letters <= 2) # 3949 - still lots of valid examples ("No!")  - but also lots of digits and abbreviations as seen above
test <- sent %>% filter(letters == 0) # All stuff that does not / cannot be translated
test <- sent %>% filter(letters == 1) # 458 - these are the initials - seems to be the sweet spot - try detecting languages ananything with letters >1
test <- sent %>% filter(letters < 2) # 3066 - looks about right - all-non translatable stuff 

# Sentences per speech
hist(spe.sent$sentences)
test <- sent %>% filter(id %in% spe.sent$id[spe.sent$sentences > 50]) # Looks reasonable - 198 is a good example for a multilingual speech
test <- sent %>% filter(id %in% spe.sent$id[spe.sent$sentences > 250]) # Looks reasonable

# 198- first couple of sentences contain four different languages - test example!
# 227370 - Good example for wrongly split sentence - "Es entsteht zurzeit innerhalb der Grenzen der Europäischen Union ein 29.", "Staat.".

# Except for decimal/ordered numbers/dates and abbreviations splitting looks quite good
```

The results suggest mostly correct sentence tokenization - but also indicate some errors regarding single letter abbreviations or wrongly split decimal numbers.

#### Local language detection

In order to avoid sending text to the translation API that is already in English (and for which no money should be spent, accordingly) we then ran three local language detection algorithms (circling around the tokenization errors note above):

Code

```
# Language detection packages
library(fastText)        # CRAN v1.0.3
file_ftz = system.file("language_identification/lid.176.ftz", package = "fastText") # The small pre-trained model
library(cld2)            # CRAN v1.2.4
library(cld3)            # CRAN v1.5.0

# Language detection ####
# Circling around potential errors along the letter limit developed above

# fastText
sent$lang_ft <- NA
sent$lang_ft[sent$letters > 1] <- fastText::language_identification(input_obj = sent$sentence[sent$letters > 1],
                                                                     pre_trained_language_model_path = file_ftz,
                                                                     k = 1,
                                                                     th = 0.0,
                                                                     threads = 1,
                                                                     verbose = T)[[1]] # This one takes a bit ...
# Compact language detector 2
sent$lang_cld2 <-NA
sent$lang_cld2[sent$letters > 1] <- cld2::detect_language(sent$sentence[sent$letters > 1])

# Compact language detector 3
sent$lang_cld3 <-NA
sent$lang_cld3[sent$letters > 1] <- cld3::detect_language(sent$sentence[sent$letters > 1])


# How often do they agree?
sent$lang_agree <- (sent$lang_cld2 == sent$lang_cld3 & sent$lang_ft == sent$lang_cld2 &  sent$lang_ft == sent$lang_cld3)
sent$lang_agree <- ifelse(is.na(sent$lang_ft) & is.na(sent$lang_cld2) & is.na(sent$lang_cld3),
                          T,
                          sent$lang_agree)
sent$lang_agree[is.na(sent$lang_agree)] <- F
sum(sent$lang_agree)/nrow(sent) # ~96%, nice

# Where do they disagree?
test <- sent %>% filter(!lang_agree)
# All cases in which either of the clds fails to provide an answer or is markedly off 
# fastText is clearly the best 

# -> Translate everything that is not "en" according to fastText and that has more than 1 letter

# Other cross-checks
test <- sent %>% filter(is.na(lang_ft)) # Good!
test <- sent %>% filter(lang_ft != "en" & letters > 1) # Stuff to be translated - Looks good!
test <- sent %>% filter(!(lang_ft != "en" & letters > 1)) # Stuff that doesn't need to be translated - Looks good, except a few short sentences wrongly calssified as English ("Pozdrawiam.", "Fru talman!", "Formand!")
test <- sent %>% filter(!(lang_ft != "en" & letters > 1) & !lang_agree) # closer look on potentially false negatives - 63843 -- all very short, the overarching majority true English 


# Mark translation need ####
# Based on the above insights
sent$toBeTranslated <- (sent$lang_ft != "en" & sent$letters > 1)
sum(sent$toBeTranslated) # 1423824   


# Cost estimation ####

# Number of characters to be sent to the API
characters <- sum(nchar(sent$sentence[sent$toBeTranslated]))
# In Dollars - 20$ for 1 Mio characters
dollars <- (characters/1000000)*20
# In Euro - today: 0.94€ = 1$
euros <- dollars*0.94
round(euros, 2) # 4238.62€


# Export sentence level data for translation ####

# Unique sentence id!
sent$sentence_id <- 1:nrow(sent)

# Export
write_rds(sent, "./data/EP_speeches_PLS-SentenceTokenizedForTranslation.rds")
```

#### Machine translation

Then we submitted all non-English sentences containing more than one letter to the Google Translation API (which detects the respective source language on its own), using the googleLanguageR package as well as a private API-key pointing for billing in the OPTED budget (handled by the WZB Berlin/Seibert Media GmbH). To ensure control of progress, catch potential errors, and minimize API overload the code proceeds sentence-by-sentence. The subsequent code ran between September 20 and 23, 2023.

Code

```
# Packages #####
library(tidyverse)       # CRAN v2.0.0
library(progress)        # CRAN v1.2.2
library(googleLanguageR) # CRAN v0.3.0
gl_auth("./private/opted-wp5-translation-4abef9cc0428.json") # Private API key linked to OPTED budget ....

# EP speeches ####
# Sentence tokenized and with language detection etc.
sent <- read_rds("./data/EP_speeches_PLS-SentenceTokenizedForTranslation.rds")


# Translation ####

# To keep track of potential errors and minimize the amount of data/costs to be burned at once
# I decided to proceed iteratively, calling the APi only when a sentence is to be translated (non-English, more than one 'letter', see earlier script)
# and only when the translated column is not populated yet (this way the loop can be run recursively, but not the column initialization!)

# ATTENTION WHEN REPEATING THE LOOP
sent$lang_Google <- NA
sent$translatedText <- NA


# # Sample for testing
# sent <- sent %>%
#   sample_n(size = 100)


# API translation sentence by sentence (if applicable)

pb <- progress_bar$new(total = nrow(sent)) # Progress
for(i in 1:nrow(sent)) {
  
  # Test if current row/sentence needs to be translated
  if (!sent$toBeTranslated[i]) {next}
  if (!is.na(sent$translatedText[i])) {next}  
  
  # Send current sentence to Google API and collect response
  tr <- gl_translate(
    sent$sentence[i],
    target = "en",
    format = "text",
    source = "", # automatic language detection
    model = "nmt")

  # Store API response in sentence data
  sent$translatedText[i] <- tr$translatedText[1]
  sent$lang_Google[i] <- tr$detectedSourceLanguage[1]
  
  # Update progress
  pb$tick()

}

gc()


# Export sentence-level results ####
write_rds(sent, "./data/EP_speeches_PLS-Sentences_Translated.rds")
```

The resulting data contain the translated text in `sent$translatedText` and, for cross-checks, the language that the Google model itself has detected in the original speech/sentence text in `sent$detectedLanguage` (almost perfectly consistent with the local fastText results).

#### Re-aggregation to speech level

The we re-assemble the individual sentences (translated or not) to the level of the individual speech, adding the a logical indicator `speeches$translationInSpeech`for whether any actually machine-translated material is contained in the `speeches$translatedText` column. Then we append these results to the original ParlLawSpeech collection of EP speeches and store the final data set.

Code

```
# Aggregate to speech level####

# Store speech ids in which translation occurred 
tr_speeches <- sent %>% 
  filter(toBeTranslated) %>% # Only speeches which at least one translated sentence
  select(id) %>% 
  unique() %>% 
  pull() # Vector of respective speech ids


# Aggregate sentences to speech text
speeches <- sent %>% 
  mutate(text = ifelse(is.na(translatedText), # If there is no translation
                       sentence,              # Keep the raw text, else ...
                       translatedText)) %>%   # Keep the translation
  group_by(id) %>%                            # Speech level grouping
  summarise(translatedText = paste(text, collapse = " ")) # Sentences separate by exactly one white-space

# Mark speeches in which translations occurred  
# Along the ids identified above
speeches <- speeches %>% 
  mutate(translationInSpeech = ifelse(id %in% tr_speeches, T, F))
sum(speeches$translationInSpeech)


# Export speech level results ####
# after merging them with the original meta data 

# Original speech level data
org_speeches <- read_rds("./data/EP_speeches_PLS-original.rds") 

# Join translations and original data
speeches <- speeches %>% 
  left_join(org_speeches, by = "id")

# # of obs of original data?
nrow(speeches) == 509649 # TRUE

# Write this to disk
write_rds(speeches, "./data/EP_speeches_PLS-Translated.rds")
```

#### Result

The resulting data file contains all data from the original ParlLawSpeech collection of ***509,649 EP speeches between July 20 1999 and April 18 2019, of which 240,932 contain (partially) machine translated text.***

## Hungarian parliament (Országgyűlés)

#### Machine translation

After prior local language detection checks we could confirm that multilingualism does hardly occur in the Hungarian parliament, so that we could operate on the *level of full speeches* in this instance. This, however, meant larger and thus slower API calls while the overall corpus is also much larger than the material that had to be translated for the EP. In total, we submitted around 1 billion characters of Hungarian speech text for machine translation into English.

The code below was initiated on September 25, 2023 and had to be partially re-run on September 26 and 27 for a subset of non-translated speeches as the texts of four unique `speech_id` exceeded the length quota of the Google API (documented in the code)

Code

```
# Packages & auth #####
library(tidyverse)       # CRAN v2.0.0
library(progress)        # CRAN v1.2.2
library(googleLanguageR) # CRAN v0.3.0
gl_auth("./private/opted-wp5-translation-4abef9cc0428.json") # Private API Key to OPTED money ...


# Dropbox path
dp <-"C:/Users/rauh/Dropbox/OPTED datasets" # WZB


# Hungary - speech corpus ####
# Validated PLS version dated from August 24 2023
speeches <- read_rds(file = paste0(dp, "/Hungary/Corpus_speeches_hungary.rds"))
speeches$tr_id <- 1:nrow(speeches) # To have at least one unique id


# Cost estimation ####
speeches$characters <- nchar(speeches$text)
characters <- sum(speeches$characters, na.rm = T)
dollars <- (characters/1000000)*20 # 20 Dollars per 1 mio characters
euros <- dollars * 0.94 # As of September 25 2023
euros # 20145.31 €
rm(characters, dollars, euros)


# Some checks ###
test <- speeches %>% filter(is.na(characters)) # Two empty chair speeches with missing link? - catch that before translating
test <- speeches %>% filter(characters < 10)  # 0
test <- speeches %>% filter(characters < 50)  # 6844 - looks legit - ELNÖK - note, however, that speaker names are in speech text ...
rm(test)
gc()


# Translation ####

# ATTENTION WHEN REPEATING THE LOOP
speeches$lang_Google <- NA
speeches$translatedText <- NA


# # Sample for testing
# speeches <- speeches %>%
#   sample_n(size = 10)

# Re-load partially translated corpus
speeches <- read_rds("./data/HU_speeches_PLS-Translated_backup.rds")
sum(is.na(speeches$translatedText)) # 37272 

# Cost estimation
test <- speeches %>% filter(is.na(translatedText))
characters <- sum(test$characters, na.rm = T)
dollars <- (characters/1000000)*20 # 20 Dollars per 1 mio characters
euros <- dollars * 0.94 # As of September 25 2023
euros # 2562 €
rm(test)
gc()


# API translation speech-by-speech (if applicable)

rm(i)
pb <- progress_bar$new(total = nrow(speeches)) # Progress
j = 0
for(i in 1:nrow(speeches)) {
  

  # Check if character in sentences
  if (is.na(speeches$text[i])) {next}  
  
  # Check if already translated (in case of abortion in between)
  if(!is.na(speeches$translatedText[i])){next} 
  
  # Exclude a couple of extraordinary speeches (above API quota) that duplicated several times (affects 370 obs in total, see below)
  if(speeches$speech_id[i] %in% c("20182022_102_49", "20182022_176_35", "20182022_192_33", "20182022_50_2")) {next}
  
  # Check iteration
  print(i)

  # Send current speech to Google API and collect response
  tr <- gl_translate(
    speeches$text[i],
    target = "en",
    format = "text",
    source = "", # automatic language detection
    model = "nmt")

  # Store API response in speech data
  speeches$translatedText[i] <- tr$translatedText[1]
  speeches$lang_Google[i] <- tr$detectedSourceLanguage[1]
  
  # Update progress
  pb$tick()
  
}

sum(is.na(speeches$translatedText)) 

gc()


# Export ####
write_rds(speeches, "./data/HU_speeches_PLS-Translated.rds")
```

#### Result

The resulting data file contains all data from the original ParlLawSpeech collection of ***487,877 Hungarian speeches between June 28 1994 and March 10 2022 and their English translations*** (in `translatedText`) and the source language detected by the Google API (in `lang_Google`, all Hungarian except two English, one Polish, and two Slovakian speeches).

## API usage and budget consumption

The figure below documents the number of request sent to the Google Cloud Translation API (as provided in the Google cloud Console on Sept 28, 2023) in the project described here. This includes some small prior tests (a.o. documented here) as well as the translations of the two big corpora described in the two preceding sections above.

API requests as documented in the Google cloud console on Sept. 28, 2023

The figure below then documents the budget consumed by these API calls. In total, *the machine translations documented here cost 24,894.30€*.

Budget consumption as documented in the Google cloud console on Sept. 28, 2023

The API-key used for machine translations has been deactivated on Sept. 28, 2023.

## Data and further documentation

The translated text data generated by the approach described here are currently archived at the WZB Berlin Social Science Center and will be publicly released together with the overall ParlLawSpeech collection (scheduled for early 2024).

The full scripts for the machine translation can be accessed in the corresponding GitHub repository.

For further questions, please contact Christian Rauh.
